# Eribulin in Cancer Treatment

**DOI:** 10.3390/md13085016

**Published:** 2015-08-07

**Authors:** Umang Swami, Umang Shah, Sanjay Goel

**Affiliations:** 1 University of Iowa Hospitals and Clinics, 200 Hawkins Dr, Iowa City, IA 52242, USA; E-Mail: umangtalking@gmail.com; 2 Department of Medical Oncology, Montefiore Medical Center, Albert Einstein College of Medicine, 1695 Eastchester Road, Bronx, NY 10461, USA; E-Mail: umshah@montefiore.org

**Keywords:** breast cancer, EMBRACE, eribulin, E7389, Halichondrin B, Halaven™, microtubule inhibitor, NSC 707389

## Abstract

Halichondrin B is a complex, natural, polyether macrolide derived from marine sponges. Eribulin is a structurally-simplified, synthetic, macrocyclic ketone analogue of Halichondrin B. Eribulin was approved by United States Food and Drug Administration in 2010 as a third-line therapy for metastatic breast cancer patients who have previously been treated with an anthracycline and a taxane. It has a unique microtubule dynamics inhibitory action. Phase III studies have either been completed or are currently ongoing in breast cancer, soft tissue sarcoma, and non-small cell lung cancer. Phase I and II studies in multiple cancers and various combinations are currently ongoing. This article reviews the available information on eribulin with respect to its clinical pharmacology, pharmacokinetics, pharmacodynamics, mechanism of action, metabolism, preclinical studies, and with special focus on clinical trials.

## 1. Introduction

**Figure 1:**
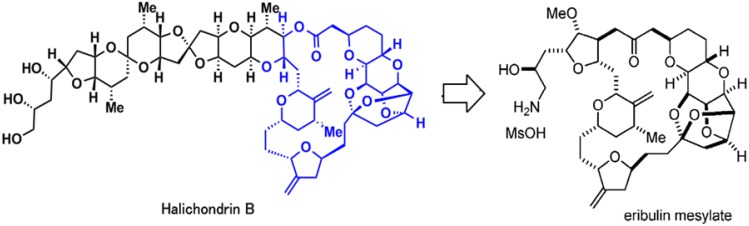
Chemical structures of Halichondrin B and eribulin mesylate.

Halicohondrin B is a natural occurring, large polyether macrolide which was originally isolated from a rare marine Japanese sponge *Halichondria okadai* Kadota in 1985 and later from other more common sponges belonging to *Axinella*, *Phakellia*, and *Lissodendoryx* families [1,2,3,4,5]. It was one of the first agents to be tested and was compared with other known antimitotic and anticancer agents using United States National Cancer Institute’s 60-cell line screen [6,7]. However, even after the confirmation of its potent anticancer activity [6,8] further development was stalled due to lack of its procurement in sufficient quantities from marine sponges. With the development of a completely synthetic method by Dr. Yoshito Kishi in 1998, and with the discovery that its cytotoxicity was a function of the macrocyclic lactone C1-C38 moiety, the drug got a new lease on life [7,9]. Thereafter, Eisai Research Institute licensed the technology and accomplished the synthesis and future development of the resulting drug, eribulin mesylate (Halaven^®^, also known as eribulin mesilate, INN codename E7389, and before that, ER-086526 and B1939, US NCI designation NSC-707389) [10,11]. The structures of Halichondrin B and eribulin mesylate are given in Figure 1.

Eribulin mesylate is a novel, completely synthetic, structurally-simplified, non-taxane, microtubule dynamics inhibitor, macrocyclic ketone analogue of Halichondrin B (NSC 609395) [11,12,13]. Due to the novel mechanism of action of eribulin, which was distinct from other known antitubulin agents, and its impressive preclinical activity, it was presented to NCI, Drug Development Group in 1998 and entered phase I clinical trials in 2002. After impressive results in phase III trials [14] the U.S. Food and Drug Administration approved eribulin on 15 November 2010, for treatment of patients with metastatic breast cancer (MBC) who havd previously received an anthracycline and a taxane in either the adjuvant or metastatic setting, and at least two chemotherapeutic regimens for the treatment of metastatic disease [15]. The recommended dose of eribulin mesylate 1.4 mg/m^2^ is equivalent to eribulin 1.23 mg/m^2^ (expressed as free base) intravenously over 2 to 5 min on days one and eight of a 21-day cycle [15,16]. However, in text for the fluency of reading we have substituted eribulin mesylate with eribulin.

Eribulin has undergone phase III clinical trials in MBC, soft-tissue sarcoma, and non-small cell lung cancer (NSCLC). Phase II trials with eribulin have been conducted or are currently ongoing as single agent, or in combination with other agents, in breast, NSCLC, salivary gland, pancreatic, prostate, head and neck cancer, bladder/urothelium and kidney dysfunction, and ovarian and related gynecological malignancies [17]. The purpose of this article is to review the available information on eribulin with special focus on clinical studies. The article also briefly reviews the preclinical information, mechanism of action, and pharmacokinetics of eribulin.

## 2. Preclinical Studies

Eribulin has shown to inhibit cell growth at sub to low nmol/L IC_50_ values (0.09–9.5 nmol/L) in *in vitro* studies, in a diverse variety of human cancer cell lines, like MDA-MB-231, -435, -468, and HCC1806 breast cancers, DU 145 and LNCaP prostate cancers, HT-29, COLO 205 and DLD-1 colon cancers, H23, H441, H520, and H522-T1 NSCLC, NCI-H82 small cell cancer, U937 histiocytic lymphoma, FaDu pharyngeal squamous cell carcinoma, A2780/1A9 ovarian cancer, MES-SA uterine sarcoma, LOX melanoma, and HL-60 promyelocytic leukemia [7,11,18].

Eribulin was found to be around 2–4 times more potent than paclitaxel and vinblastine in inhibiting growth of different cancer cell lines and similar to Halichondrin B. Also eribulin did not show cytotoxic effects even with higher concentrations against dormant IMR-90 human fibroblasts, indicating growth inhibition by low levels of eribulin is specific for proliferating cells and not due to nonspecific cytotoxicity [7]. In NSCLC cell lines Calu-1 (p53-null) and A549 (p53 wild type) eribulin showed p53-independent anticancer activity in the 0.5 pM range [19]. Though eribulin showed similar activity as Halichondrin B [7,18], it was more potent in its interactions with tubulin in *in vitro* [20] and *in vivo* studies, as well as less toxic, as seen in granulocyte-macrophage colony forming units [21]. 

In *in vitro* studies on SK-BR-3 cell lines eribulin showed synergistic activity with many drugs like gemcitabine, cisplatin, epirubicin, trastuzumab, docetaxel, and vinorelbine and additive effects with carboplatin and antagonistic effects with 5′-DUFR [22]. Antagonism with carboplatin was seen in some NSCLC cell lines while additive response in others [18]. Upon estrogen-stimulation, eribulin showed potent antitumor effects on estrogen receptor positive (ER+) breast cancer cells, whereas the combined treatment of eribulin with an antiestrogen resulted in a weakly-additive antitumor effect. Eribulin seems to exhibit anticancer stem cell effects on both ER+ and negative breast cancer cells [23]. Eribulin alone, or combined with RAD001, a mTOR inhibitor, showed cell growth inhibition in triple-negative breast cancer and HER2 cell lines, dose-related inhibition of Akt activation, significant synergistic growth inhibition with combination treatment, and reversal of the pAkt feedback response with mTOR inactivation [24]. Eribulin retained full *in vitro* potency in cells harboring beta-tubulin mutations that leads to substantial resistance to taxanes, as seen in paclitaxel-resistant ovarian cancer sublines [25]. Eribulin has shown to inhibit platinum-resistant ovarian cancer cell lines, like RMG-I, PEO23, and PEO4, which also have high human telomerase reverse transcriptase expression. Eribulin directly inhibited RNA-dependent RNA polymerase activity, but not telomerase activity, of human telomerase reverse transcriptase *in vitro*, which might explain its activity in this setting [26].

In isolated squid axoplasm eribulin has shown to inhibit anterograde fast axonal transport, with the potency being vincristine = ixabepilone > paclitaxel = eribulin [27]. However, in contrast to vincristine and ixabepilone, eribulin and paclitaxel did not inhibit retrograde fast axonal transport and had insignificant effects on an *in vitro* microtubule gliding assay consisting of recombinant kinesin (kinesin-1) and microtubules composed of purified bovine brain tubulin. These results suggest that inhibition of microtubule-based fast axonal transport may be a significant contributor to neurotoxicity induced by these agents, and different classes of drugs may cause it through different mechanisms [27]. 

Eribulin demonstrated tumor regressions, remissions, and increased lifespan at dose levels below the maximum tolerated dose (MTD) in *in vivo* breast, ovary, colon, lung, melanoma, pancreatic, and fibrosarcoma human tumor models in mice [7,11]. As compared to paclitaxel (run at empirically determined MTD levels), eribulin showed significant and superior *in vivo* anticancer efficacy in MDA-MB-435, COLO 205, and LOX cell lines (in NIH:OVCAR-3 model, significant only) at much lower doses [7]. Eribulin also showed a much wider *in vivo* therapeutic window as compared to paclitaxel (five-fold *vs.* <2.0 in LOX and four-fold *vs.* 1.7 in MDA-MB-435 models), which can lead to the possibility of increasing the dosage above a fully tumor-suppressive dose, which can subsequently lead to more complete tumor eradication and can explain its superiority over paclitaxel [7,18].

Dose scheduling studies with eribulin on MDA-MB-435 breast, HT-1080 fibrosarcoma, U251 glioblastoma, SR-475 head and neck cancer, SK-LMS-1 leiomyosarcoma, NCI-H322M and NCI-H522 NSCLC, PANC-1 pancreatic cancer, and NCI-H82 small cell lung cancer (SCLC) models showed that maximal efficacy and minimal toxicity is achieved with moderate intermittent dosing [21,28,29]. Eribulin, at a dose of 0.1–0.4 mg/kg and q4d × 3 schedule, which has a limited tumor inhibitory effect, when combined with gemcitabine 120–270 mg/kg and q3d × 4 schedule, which has only tumor stasis effect, induced significant regression in H522 NSCLC xenografts [28]. However eribulin in combination with doxorubicin was not synergistic in the MDA-MB-435 xenograft model [18,28]. In a pediatric preclinical testing program *in vivo* xenograft panels eribulin, at a dose of 1 mg/kg (solid tumors), or 1.5 mg/kg (ALL models), using a q4d × 3 schedule repeated at day 21 was well tolerated. Eighteen of 35 (51%) solid tumor xenografts showed objective responses. Complete responses (CR) or maintained CR were observed in panels of Wilms tumor, rhabdomyosarcoma, Ewing sarcoma, glioblastoma, osteosarcoma, and all eight acute lymphocytic leukemia (ALL) xenografts. Eribulin induced significant differences in event-free survival distribution in 29 of 35 (83%) of the solid tumors and in 8 of 8 (100%) of ALL xenografts, as compared to controls. In solid tumor panels, eribulin was found to be equal or superior to that observed previously with vincristine. [30]. 

Eribulin showed less neurotoxicity in female BALB/c mice, as compared to paclitaxel or ixabepilone at equivalent MTD-based doses [31]. As compared to additional paclitaxel treatment, eribulin also showed a reduced tendency to exacerbate preexisting paclitaxel-induced polyneuropathy [32].

## 3. Mechanism of Action

**Figure 2:**
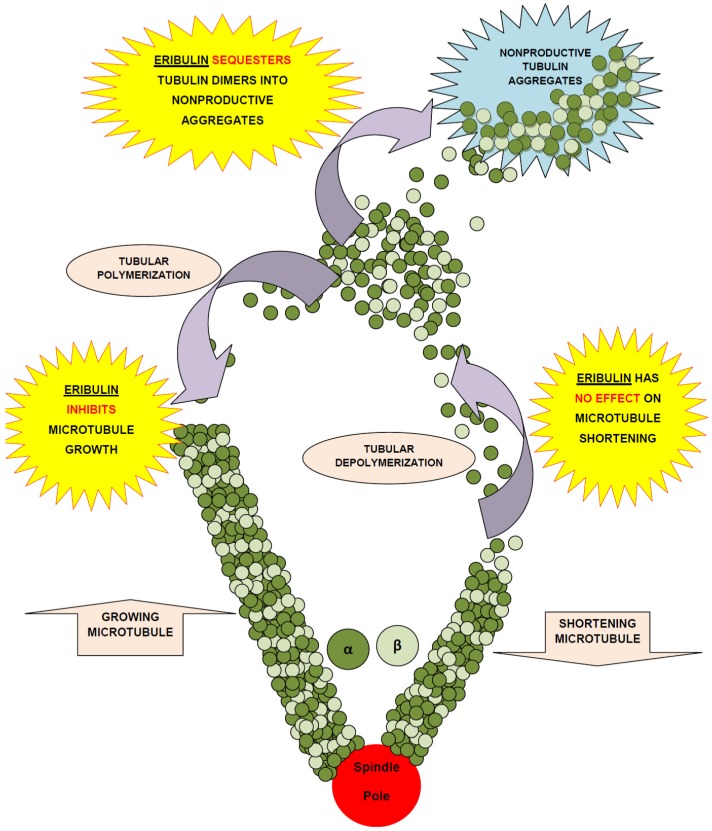
Mechanism of action of eribulin mesylate.

Eribulin is a simplified macrocyclic ketone in which the C1 lactone ester of Halichondrin B is replaced by ketone, the tricyclic C29–38 system is replaced by a single five membered ring, C31 methyl is replaced by methoxy, and the entire C39–C54 polyether side-chain is removed. [11,15]. Although eribulin is considered as an antitubulin drug, it inhibits microtubule dynamics via a novel mechanism of action [20,33,34], which seems to involve binding to a unique site on tubulin [20], resulting in the suppression of microtubule polymerization rather than shortening, without affecting depolymerization along with sequestration of tubulin into nonfunctional aggregates [7,33,35,36] (Figure 2). 

Eribulin has an end poisoning mechanism. It may suppress mitosis by either directly binding as un-liganded eribulin to microtubule ends or by competing with un-liganded soluble tubulin for addition to growing microtubule ends by inducing tubulin aggregates, leading to the formation of abnormal mitotic spindles which cannot pass the metaphase/anaphase checkpoint. It causes microtubule growth inhibition and tubulin sequestration into non-functional aggregates [33]. Eribulin seems to inhibit tubulin polymer formation by binding to either the interdimer interface or the β-tubulin subunit alone. It does not bind to both α- and β-tubulin [37,38]. At 100 nM (eribulin concentration that inhibits microtubule plus end growth by 50% or the concentration approximately 10 times higher than that minimally induces complete G2/M blocks) it suppresses dynamic instability by binding, with high affinity, at microtubule plus ends but does not suppress dynamic instability at microtubule minus ends [11,37]. It inhibits cancer cell growth via induction of irreversible complete mitotic block at G_2_-M (prometaphase blockage), disruption of mitotic spindles formation, and initiation of apoptosis following prolonged mitotic blockage [7,39]. 

Studies suggest that tumors expressing higher levels of βIII tubulin isotype may be more responsive to eribulin [40]. It is interesting, as the βIII tubulin gene is found to confer resistance to [41] and is inducible by [42] various antitubulin agents, like vinorelbine and paclitaxel. Additionally, its over-expression is correlated either with low response rates in patients treated with regimens containing taxanes or vinorelbine or with reduced survival in patients with NSCLC, breast, gastric, ovarian, and cancers of unknown primary site [43]. 

Eribulin seems to reverse epithelial mesenchymal transition by downregulating transforming growth factor-β induced Smad phosphorylation which may contribute to decreased metastasis [44]. Eribulin has also shown inhibitory effects on Wnt/β-catenin signaling when tested *in vitro* and *in vivo* on small bowel adenocarcinoma cell line, SIAC1 [45]. 

Eribulin has shown to induce similar changes in gene expression in human umbilical vein endothelial cells (HUVEC) as paclitaxel with majority (59%) of genes overlapping for both treatments. However, in human brain vascular pericytes (HBVP) altered gene expressions was drug-specific and overlap was only 12%. A significant upregulation of NOTCH3 expression was detected after eribulin treatment in HBVP. Eribulin, but not paclitaxel, caused dramatic shortening and interruption of pericyte-driven capillary networks in HUVEC and HBVP co-culture assay at low nmol/L concentrations, which indicates eribulin’s potent antiangiogenic effect against pericyte-driven *in vitro* angiogenesis in addition to its cytotoxicity [46]. 

Eribulin has shown to induce tumor vascular remodeling in human breast cancer MX-1 and MDA-MB-231 xenograft models, as observed by dynamic-contrast enhanced MRI in nude rats through novel antivascular activity. On Hoechst 33342 staining it appeared to increase tumor vascular perfusion by vascular remodeling and on CD31 immunohistochemical staining it showed to increase microvessel density along with decrease mean vascular areas and fewer branched vessels in tumor tissues. On quantitative RT-PCR gene expression profiling eribulin appeared to alter gene expression in angiogenesis and epithelial mesenchymal transition-related signaling pathways within the abnormal tumor stroma. Eribulin also decreased expression of mouse vascular endothelial growth factor protein levels and human CA9 protein, which indicates a reduction in degree of hypoxia in tumor xenograft models. In MDA-MB-231 xenograft model, prior eribulin treatment showed to enhance anti-tumor activity of capecitabine. Thus, it appears that eribulin induces tumor vasculature remodeling in breast cancer models, leading to increased perfusion and decreased hypoxia which might lead to better penetration of subsequent anticancer agents and subsequent enhanced antitumor activity [47]. 

## 4. Pharmacokinetics and Metabolism

### 4.1. Pharmacokinetics

**Figure 3:**
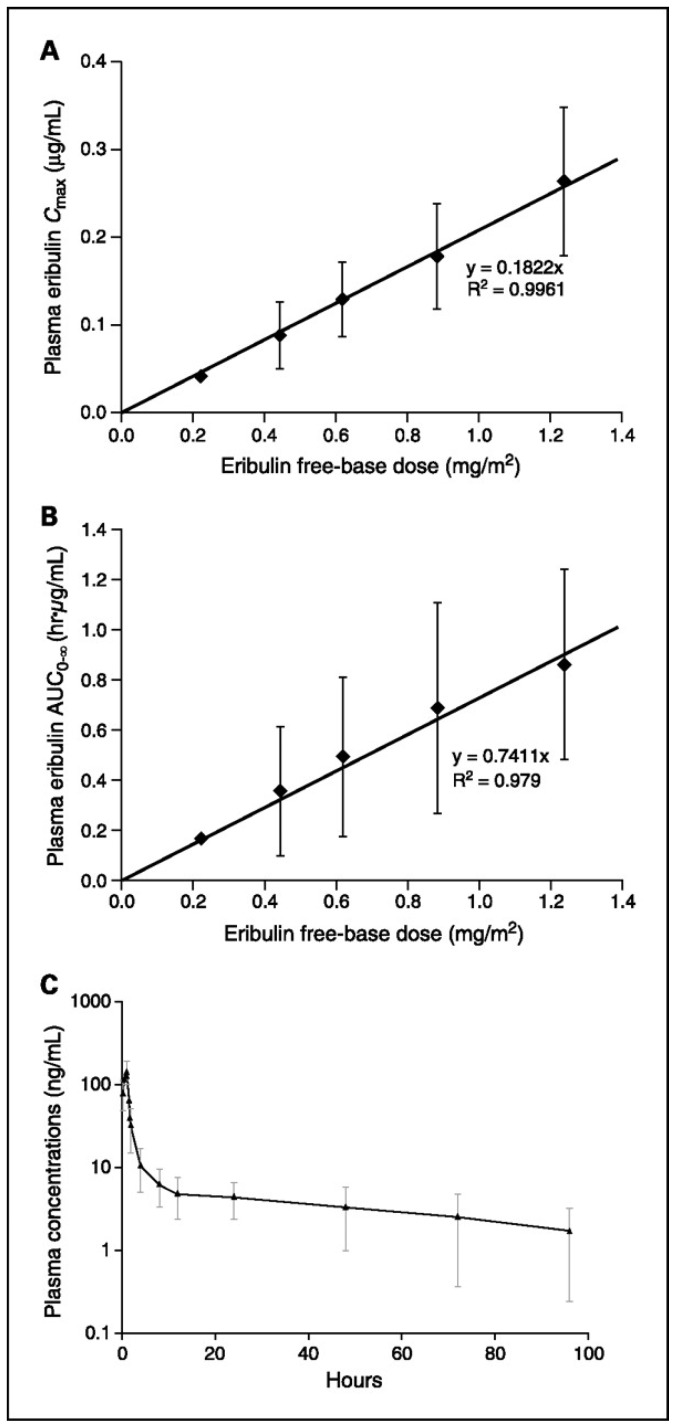
(**A**) Plasma Cmax *versus* dose following a 1-h infusion of eribulin on day 1; (**B**) Plasma area under the concentration-time curve (AUC)_(0–∞)_ *versus* dose following a 1-h infusion of eribulin on day 1; (**C**) Plasma concentration *versus* time profile for the 1.0 mg/m^2^ treatment group (*n* = 9) [50].

The pharmacokinetics results from first phase I trial using eribulin bolus every three weeks out of four demonstrated a tri-phasic elimination with prolonged terminal half-life of 36–48 h. Eribulin levels in plasma at MTD were above concentrations required for *in vitro* cytotoxicity for >1 week. After 48 h around 10% of the dose was recovered in the urine. [48,49]. Eribulin pharmacokinetics (with 1 h infusion) is linear and dose-proportional over the dosing range of 0.25–1.4 mg/m^2^ (Figure 3). Its pharmacokinetic parameter estimates between the first and third i.v. doses (days 1 and 15) at each dose level are consistent. The plasma concentration-time profile demonstrates a rapid distribution phase with a mean distribution (half-life of ~0.43 h) followed by a slower elimination phase (half-life of 38.7 h) [50].

Hepatic impairment seems to decrease clearance, prolong elimination half-life and eribulin exposure. In a phase I study on patients with liver dysfunction, eribulin was generally safe and well-tolerated. Results are summarized in Table 1. The mean dose-normalized C_max_ of eribulin was similar in the Child-Pugh A (1.15-fold; 90% Confidence Interval (CI) 0.81–1.63) and Child-Pugh B group cohort (1.29-fold; 90% CI: 0.89–1.89) as compared to normal hepatic function. The mean dose-normalized area under the curve (AUC)_(0–∞)_ increased 1.75-fold (mild) (90% CI: 1.15–2.66) in the Child-Pugh A and 2.48-fold (90% CI: 1.57–3.92) in the Child-Pugh B cohort, when compared to the normal hepatic function group [51]. Due to this, a lower starting dose is recommended in patients with mild (Child-Pugh A) and moderate (Child-Pugh B) hepatic impairment [15].

**Table 1:** Eribulin pharmacokinetics comparison in normal, mild, and moderate hepatic impairment [51].

	Normal Hepatic Function	Mild Hepatic Impairment (Child-PughA)	Moderate Hepatic Impairment (Child-Pugh B)
Safety population	6	7	5
Dose on Day 1(mg/m^2^) i.v.	1.4	1.1	0.7
Clearance (L/h)	4.57	2.75	2.06
Elimination half life (h)	36.1	41.1	65.9
C_max_ (ng/mL)	186	147	113
Mean dose normalized C_max_ (ng/mL/mg)	72	83.9	100
Mean dose normalized AUC_(0–**∞**)_ (ng·h/mL/mg)	229	420	646

AUC_(0–∞)_: Area under the concentration time curve from zero (pre-dose) extrapolated to infinity; C_max_ Maximum observed plasma concentration.

Renal impairment decreases eribulin clearance and increases eribulin exposure. In a phase I study, 19 patients with advanced and refractory solid tumors were enrolled to evaluate the effect of renal dysfunction on eribulin pharmacokinetics. Patients were grouped according to the Cockcroft-Gault formula under moderate impairment (*n* = 7, creatinine clearance [CrCl]: 30–50 mL/min), severe impairment (*n* = 6, CrCl: 15–29 mL/min), or matched-normal (*n* = 6, CrCl: ≥80 mL/min). During cycle 1, eribulin dose was 1.4 mg/m^2^ and 1.1 mg/m^2^ on days 1 and 8 respectively for moderate; 0.7 mg/m^2^ on days 1 and 8 for severe and 1.4 mg/m^2^ on days 1 and 8 for normal. The dose for severely impaired patients was based on an interim pharmacokinetic analysis of the moderate group. Moderate and severe renal impairment has an increased mean dose-normalized AUC as compared to patients with normal renal function (ratio for both: 1.49; 90% CI: 0.9–2.45). With moderate impairment dose normalized maximum plasma concentration increased by 1.31 fold (90% CI: 0.84–2.05), and with severe impairment, by 2.02-fold (90% CI: 1.27–3.21). Similar toxicities and no unexpected adverse events were observed in groups. A positive correlation was observed between clearance and renal function on regression analysis with a numerically small slope (0.0184; 90% CI: 0.00254–0.0394), indicating a small effect of renal impairment on eribulin disposition. Similar exposure with 1 mg/m^2^ with moderate and severe renal impairment is expected as with 1.4 mg/m^2^ in patients with normal renal function, based on simulations of expected AUC values. Pharmacokinetic evaluation supports dose reduction to 1 mg/m^2^ with moderate and severe renal impairment [52].

To study the effect of CYP3A4 inhibitors, a randomized, open-label, two treatments, two sequences, crossover phase I study with ketoconazole and eribulin was conducted in patients with advanced solid tumors [53]. Group 1 received 1.4 mg/m^2^ eribulin (day 1), followed by 0.7 mg/m^2^ eribulin plus 200 mg ketoconazole (day 15), and 200 mg ketoconazole alone (on day 16) of a 28-day cycle. Group 2 received 0.7 mg/m^2^ eribulin plus 200 mg ketoconazole (day 1) and 200 mg ketoconazole (day 2), followed by 1.4 mg/m^2^ eribulin (day 15) of a 28-day cycle. Eribulin dose with ketoconazole was reduced to half due to safety concerns. Ten patients (four in group one, six in group 2) were evaluable for pharmacokinetic sampling, which was performed up to 144 h following administration of eribulin. Ketoconazole had no effect on single dose exposure to eribulin (ratio of geometric least square means: AUC_(0–∞)_ = 0.95, 90% CI: 0.80–1.12 and C_max_ = 0.97, 90% CI: 0.83–1.12). Ketoconazole showed no effect on elimination half-life and clearance of eribulin [53]. To study the effect of CYP3A4 inducers, an open-label, non-randomized phase I study was conducted with rifampicin and eribulin in patients with advanced or refractory solid tumors [54]. Eribulin (1.4 mg/m^2^) was administered on days 1 and 15 and oral rifampicin 600 mg on days 9 to 20 of a 28 day cycle. Subsequently, patients were allowed to continue eribulin on days 1 and 8 of a 21 day cycle. Eleven patients were evaluable for pharmacokinetic analysis, which was performed up to 144 h following drug administration. Rifampicin had no effect on single dose exposure to eribulin (geometric least square means ratio: AUC_(0–∞)_ = 1.10, 90% CI: 0.91–1.34, C_max_ = 0.97, 90% CI: 0.81–1.17) [54]. Therefore, CYP3A4 inhibitors and inducers seem to affect eribulin exposure [53,54]. 

In a phase II study eribulin (1.4 mg/m^2^ on days 1 and 8 of 21 day cycle) pharmacokinetics was evaluated in heavily-pretreated, locally-advanced or MBC patients. As seen above, in other studies, eribulin pharmacokinetics was best described by a three-compartment model and slow elimination from a central compartment. In a typical patient with adequate organ functions, clearance was 2.98 L/h, central volume of distribution was 3.72 L, volumes of two peripheral compartments were 3.60 L and 126 L, and inter-compartmental clearances were 2.7 L/h and 5.6 L/h, respectively. The inter-patient variability in clearance was 57% and ranged 26%–98% for other parameters with a 21% residual error (proportional). In patients with elevated AST, clearance was lower on an average of 38% and positively correlated with the renal function. Appreciable interpatient pharmacokinetic variability was observed, a minor fraction of which was explained by measures of liver and renal function [55]. 

### 4.2. Metabolism

A phase I study on six patients with advanced solid tumors using [^14^C] eribulin acetate did not find any major metabolite of eribulin in plasma. The elimination half-life of eribulin (45.6 h) was comparable to total radioactivity (42.3 h). Eribulin was primarily eliminated unchanged in feces, while urine constituted a minor elimination route [56]. 

Eribulin is a substrate for P glyocoprotein (P-gp) drug efflux pump, leading to reduced *in vitro* activity against multidrug-resistant cells which over-express P-gp drug efflux pump [57]. In intact and bile duct cannulated rats and dogs the unchanged drug was found to be the major component in plasma, bile, urine, and feces following i.v. dosing. Eribulin is not strongly bound to mouse, rat, dog or human plasma protein with an interspecies difference. This suggests that variability in albumin or α1-acid glycoprotein will not significantly affect eribulin pharmacokinetics [11,18].

In *in vitro* studies CYP3A4 appears to be the major enzyme responsible for the human hepatic metabolism of eribulin [11]. No significant inhibition of CYP1A2, CYP2C9, CYP2C19, CYP2D6, or CYP2E1 was detected with eribulin at concentrations up to 5 μM [11]. Similar to terfenadine, eribulin acts as a CYP3A4 substrate competitor and not as a mechanism-based inhibitor. It does not induce CYP1A and CYP3A expression or activities [58]. Although eribulin competitively and reversibly inhibits nifedipine dehydration, testosterone 6-β-hydroxylation and *R*-warfarin 10-hydroxylation activities of recombinant CYP3A4, it does not induce or inhibit hepatic CYP3A4 activity nor does it inhibit CYP3A4-mediated metabolism of various therapeutic agents, including tamoxifen, paclitaxel, midazolam, carbamazepine, diazepam or terfenadine, at clinically relevant concentrations [18,58]. Therefore, eribulin does not seem to inhibit the metabolism of concurrently-administered drugs metabolized by CYP3A4 or CYP1A, suggesting a minimal risk of drug–drug interactions [11,58].

## 5. Clinical Trials: Safety and Efficacy

### 5.1. Phase I Studies

Phase I studies of eribulin are summarized in Table 2. The first phase I trial of eribulin on patients with refractory or advanced solid tumors [48] used a rapid titration design with real-time pharmacokinetic analysis to guide dose escalation [59]. The starting dose was 0.125 mg/m^2^/week. The rapid escalation phase, with single-patient cohorts, with intra- and inter-patient dose doubling until toxicity was observed, ended with a grade (G) 3 elevation of alkaline phosphatase at a dose of 0.5 mg/m^2^/week. The second phase consisted of standard 3 + 3 dose escalation schedule, which ended at 2.0 mg/m^2^/week with two dose-limiting toxicities (DLT’s); one G 3 febrile neutropenia and one G 4 neutropenia [48]. The second phase I trial [50] followed NCI-accelerated titration scheme design 4B [59]. Thirty-two patients were treated for a median numbers of two cycles (range, 1–10). At the highest dose level of 1.4 mg/m^2^, DLT of G 4 neutropenia was seen in two patients and one of these patients also experienced G 3 fatigue. Three other patients at 1.4 mg/m^2^ experienced G 3 neutropenia (not DLT), leading to the omission of the week 3 dose in cycle 1. Therefore, the MTD in this study was regarded as 1.0 mg/m^2^. Eribulin was found to be safe, with low incidence of neuropathy (25%, all G 1/2) or cumulative toxicities and with an absence of hypersensitivity reaction [50]. Another phase I trial enrolled 21 patients with advanced solid tumors [60]. Febrile neutropenia as DLT was seen in all three patients at 4 mg/m^2^, 2/3 patients at 2.8 mg/m^2^ (on one dose reduction) and 1/7 patients treated at 2.0 mg/m^2^. Therefore MTD was established at 2.0 mg/m^2^. Seven patients experienced treatment related serious adverse events including G 4 febrile neutropenia (five patients), G 3 hyponatremia (one patient) and G 4 febrile neutropenia, G 2 pyrexia, and G 3 infection (one patient) [60]. 

A fourth single-center, dose-escalation phase I study was conducted on 15 Japanese patients. Three, three, six and three patients were treated at 0.7, 1.0, 1.4 and 2.0 mg/m^2^ drug cohorts, respectively. A median of two cycles (range 1–15) were administered. Five of 15 patients experienced neutropenia or febrile neutropenia as DLTs [61].

In phase I trial on patients with liver dysfunction, six patients experienced treatment related G ≥ 3 toxicities [51]. In a first-in-human phase I study of eribulin in patients with renal dysfunction and advanced urothelial cancer (UC), eribulin was given in tiers of 0.7, 1.0 and 1.4 mg/m^2^ on days 1 and 8, every three weeks with dose escalation in 3 + 3 design. Overall 21 patients received a median number of six cycles (range 0–16). In moderate renal dysfunction (≥40–59 mL/min, Cockroft-Gault) cohort there were no DLTs. In severe renal dysfunction (20–40 mL/min, not needing dialysis) cohort one out of six patients treated at 1.4 mg/m^2^ had a DLT of G 3 weakness and fatigue. Median progression-free survival (PFS) was 4.1 months (range 2.8–8.8) and median survival was 9.7 months (range 7.1–19.7 months) at a median follow-up of 11 months. Eribulin tolerance of full dosage with encouraging activity may fulfill an unmet need across a spectrum of cancers in patients with renal dysfunction [62]. However a lower starting dose is recommended for patients with moderate renal impairment (CrCl 30–50 mL/min) [15]. 

**Table 2:** Phase I studies of eribulin.

Study	Treatment Regimen and RPIID or MTD	Evaluable Patients	Partial Response	Stable Disease	Important Toxicities as Reported
**Single Agent**					
Synold *et al*. [48]	Weekly bolus three weeks out of four, MTD-1.4 mg/m^2^/week	38	2 (NSCLC; bladder)	Three marginal responses (NSCLC, breast, and thyroid) and 12 SD (median of 4 months; range 2–14 months)	NR
Goel *et al*. [50]	One-hour infusion on days 1, 8 and 15 of a 28-day cycle MTD-1 mg/m^2^	25	Unconfirmed (lasting 79 days) in cervical cancer. Patient progressed before her response was confirmed at the next tumor assessment.	10 (range from 39 to 234 days)	Most common-fatigue (53%), nausea (41%), and anorexia (38%).
					Eribulin related G 3/4 toxicities included neutropenia (19%), fatigue (13%), anorexia (3%), anemia (3%) and vomiting (3%).
Tan *et al*. [63]	One-hour eribulin infusion on day 1 of 21-day cycle MTD-2.0 mg/m^2^	21	Unconfirmed in NSCLC patient at 4 mg/m^2^	12 (median duration of 86 days; range 47–386 days)	Most common-neutropenia (38%), fatigue (33%), alopecia (33%), febrile neutropenia (29%), anemia (24%) and nausea (19%).
Mukohara *et al*. [61]	Bolus on day 1 and 8 of 21 days MTD-2.0 mg/m^2^ RPIID-1.4 mg/m^2^	14	3 (2 NSCLC and 1 with head and neck cancer)	4 (more than 12 weeks)	Most common G 3/4 toxicities-neutropenia (67%), lymphocytopenia (20%), febrile neutropenia (33%), and fatigue (13%).
**Organ Dysfunction and Other Miscellaneous Pharmacokinetic and Safety Studies**					
Devriese *et al*. [53]	Eribulin with oral ketoconazole (CYP3A4 inhibitor) MTD-NA	10	0	7	Most common-fatigue (66.7%), nausea (66.7%), alopecia (50%), neutropenia (42%)
Devriese *et al*. [54]	Eribulin with oral rifampicin (CYP3A4 inducer) MTD-NA	11	1 (breast cancer)	4	Most common-fatigue (64%), alopecia (50%), nausea (43%) and pyrexia (36%).
Devriese *et al*. [51]	Eribulin in liver dysfunction—A lower starting dose is recommended in patients with mild (Child-Pugh A) and moderate (Child-Pugh B) hepatic impairment	18	0	9	Most common-alopecia (67%), fatigue (39%), neutropenia (33%), nausea (28%) and vomiting (22%)
Synold *et al*. [62]	Eribulin in renal dysfunction and advanced urothelial cancer on days 1 and 8, every three weeks MTD in moderate renal dysfunction (≥40–59 mL/min, Cockrot-Gault) and severe renal dysfunction (20–40 mL/min, not needing dialysiswas 1.4 mg/m^2^)	20	3	9	G 3/4 neutropenia (five patients), febrile neutropenia (one patient), G 1 sensory neuropathy (seven patients) and G 1 transaminitis (eight patients)
Tan *et al*. [52]	Eribulin in normal and impaired renal function	NR	NR	NR	NR
	Recommendations-Eribulin dose reduction to 1 mg/m^2^ in moderate and severe renal impairment				
Lesimple *et al*. [64]	Eribulin 1.4 mg/m2, days 1 and 8 of 21 day cycle for QT assessment MTD-NA	NR	NR	NR	No proarrhythmic event
Dubbelman *et al*. [56]	Mass balance study of [^14^C]eribulin MTD-NA	NR	NR	NR	Most common-fatigue (50%)
**Combination Studies**					
Goel *et al*. [65]	Eribulin and gemcitabine	17	1 PR (ovarian cancer)	Eight SD with 4 minor responses (2 NSCLC, 1 endometrial and 1 head and neck cancer)	G 3/4 toxicities were neutropenia (six patients), leukopenia (three patients), anemia (two patients) and thrombocytopenia (two patients).
	RPIID-eribulin 1.0 g/m^2^ and gemcitabine 1000 mg/m^2^ days 1 and 8, every 3 weeks				
Koczyvas *et al*. [66]	Eribulin with cisplatin MTD/RPIID-Eribulin (1.2 mg/m^2^ on days 1 and 8) and cisplatin (75 mg/m^2^ on day 1) of 21 days cycle	36	2 unconfirmed PR (pancreatic, breast), 2 confirmed PR (esophageal and bladder)	12	Most common-neutropenia (78%), anemia (58%), and fatigue (39%).
Mukai *et al*. [67]	Eribulin with trastuzumab	12	1	10	Most common-neutropenia (100%), leukopenia (100%), anemia (66.7%) and alopecia (66.7%).
	RPIID-Eribulin 1.4 mg/m^2^ (on days 1 and 8 of a 21-day cycle) with either weekly trastuzumab (4 mg/kg loading dose, 2 mg/kg/week) or tri-weekly trastuzumab				
	(8 mg/kg loading dose, 6 mg/kg/tri-week)				
Swami *et al*. [68]	Eribulin with carboplatin	42	1 CR (tonsillar cancer) 2 PRs (prostate)	24	Most common-neutropenia (52%; 40% G 3/4), thrombocytopenia (29%; 13% G 3/4), fatigue (58%; 4% G 3/4), and nausea (40%; no G 3/4).
	RD-Eribulin (1.1 mg/m^2^ bolus, on days 1 and 8) with carboplatin (AUC 6, 30 min iv infusion on day 1) every 21 days with eribulin given first				
Ruong *et al*. [69]	Eribulin with cyclophosphamide RPIID eribulin 1.4 mg/m^2^ on day 1 and day 8 with cyclophosphamide 600 mg/m^2^ on day 1	6	2	4	All G toxicities included neutropenia (50%), thrombocytopenia, fatigue, nausea, peripheral neuropathy, rash, mucositis, alopecia (33% each), and elevated liver enzymes (17%). Only G 3/4 toxicity was neutropenia requiring G-CSF support.
Nasim *et al*. [70]	Eribulin with capecitabine	NR	NR	NR	NR
	RPIID-schedule 2 MTD-Eribulin 1.4 mg/m^2^ (days 1 and 8) with twice-daily oral capecitabine 1000 mg/m^2^ (days 1–14) every 21 days				
Vogelzang *et al*. [71]	Eribulin with gemcitabine/cisplatin RPIID-Eribulin 1.0 mg/m^2^ (days 1 and 8 of 21 day cycle with gemcitabine (1000 mg/m^2^, days 1 and 8) and cisplatin (70 mg/m^2^, day 1)	9	Two CRs (one confirmed, one unconfirmed) and six PRs (four confirmed, two unconfirmed)	NR	Most common adverse events at RPIID-nausea (83%), neutropenia (83%), fatigue (83%), thrombocytopenia (83%), anemia (83%), and anorexia (50%).
Sakiyama *et al*. [72]	Eribulin with S-1	11	5	5	G 3/4 toxicities-neutropenia (83.3%), G 3 febrile neutropenia (25.0%), hypokalemia (8.3%)
	RPIID Eribulin 1.4 mg m^2^, days 1 and 8 and S-1 65 mg m^2^ from days 1 to 14 of 21 day cycle				
Waller *et al*. [73]	Eribulin with pemetrexed MTD-Eribulin 0.9 mg/m^2^ with pemetrexed (500 mg/m^2^) each on day 1 of a 21-day cycle	NR	NR	NR	NR
**Neoadjuvant Therapy**					
Schwartzberg *et al*. [74]	Eribulin, carboplatin and trastuzumab	12	At surgery, 10 achieved PR and 2 had pathologic complete response	NA	G 3/4 hematological toxicities included anemia (41%), thrombocytopenia (33%) and neutropenia (75%)
	Not planned for phase II development				

RPIID—Recommended phase II dose; MTD—Maximum tolerated dose; CR—Complete response; PR—Partial response; SD—Stable disease; NR—Not reported; NA—Not applicable; G—Grade.

In a phase I study of eribulin and gemcitabine in patients with advanced solid tumors, 21 patients were treated for a median number of two cycles (range 1–8). The DLTs were G 3 diarrhea (one patient) and G 3 dizziness/fatigue (one patient). [65]. Combination of eribulin with cisplatin was evaluated in a phase I study in 36 patients with advanced solid tumors. Eribulin was administered on days 1, 8, and 15 of every 28 day cycle, which was later changed to eribulin on days 1 and 8 every 21 day cycle, with cisplatin administered on day 1. The change was done due to an inability to administer treatment on day 15 (due to neutropenia) and to avoid long delays with cisplatin administration. A median number of 15 (range 2–32) weeks of treatment was administered to all patients, and the median number of cycles at the MTD was 4.5 (2–8), corresponding to 19 weeks (6–28). No apparent effect of cisplatin on eribulin pharmacokinetics was observed. G 2 neuropathy due to treatment was observed in three patients. Three patients experienced DLT’s on the 28-day cycle (G 4 febrile neutropenia, G 3 anorexia/fatigue/hypokalemia; and G 3 stomatitis/fatigue) and three patients had DLTs on 21-day schedule (G 3 hypokalemia/hyponatremia, G 4 mucositis and G 3 hypokalemia) [66]. Eribulin in combination with trastuzumab was tested in a phase I study in Japanese patients with advanced or recurrent HER2+ breast cancer. The study consisted of two parts. Eribulin (1.4 mg/m^2^) was administered on days 1 and 8 of every three week cycle. In part one, trastuzumab was administered as a 4 mg/kg loading dose followed by 2 mg/kg weekly doses and in part two as 8 mg/kg loading dose followed by 6 mg/kg tri-weekly doses. Overall 12 patients (six for each regimen) received the treatment. No DLT was observed. No PK interaction was observed between eribulin and trastuzumab and the combination was well tolerated. A G 2 decrease in ejection fraction was observed in two patients with recovery after one week without treatment. Therefore, cardiac function should be routinely assessed in patients receiving the combination therapy of eribulin mesylate with trastuzumab [67].

Another phase I combination trial of eribulin was conducted with carboplatin in patients with advanced refractory solid tumors. Carboplatin was administered as a 30-min infusion and eribulin as a 2- to 5-min i.v. bolus, separated by 1 h. In stage 1, eribulin (0.7, 0.9, 1.1, and 1.4 mg/m^2^) was dose-escalated with carboplatin at a fixed AUC of 5 using 3 + 3 design in two schedules, differing by the order of administration. In stage 2, eribulin (1.1 and 1.4 mg/m^2^) was dose escalated with carboplatin at a fixed AUC of 6 using the preferred schedule from stage 1. Fifty-two patients were treated. In stage 1, a DLT of diarrhea was experienced. The MTD was defined as 1.4 mg/m^2^ with eribulin given first. In stage 2, DLTs in 1/6 patients (febrile neutropenia) at 1.1 mg/m^2^ and in 2/3 patients (febrile neutropenia, neutropenia) at 1.4 mg/m^2^, were observed defining the MTD as 1.1 mg/m^2^. One CR in tonsillar cancer and two PRs in prostate cancer were observed. The pharmacokinetic analyses suggested absence of any interaction between eribulin and carboplatin [68]. An extension arm investigated the efficacy and safety of the combination in chemo-naïve advanced NSCLC patients with measurable disease. Eribulin mesylate (1.1 mg/m^2^, on days 1 and 8) with carboplatin (AUC 6, on day 1) was given every 21 days as per recommended phase II dose. Twelve patients were enrolled and 11 patients were evaluable for efficacy. Objective response rate (ORR) was 27.3% (all PRs), disease control rate (DCR) was 63.6%, median overall survival (OS) was 12.1 months (range 1.6–12.1, five patients still alive at the time of study presentation); PFS was 4.2 months (0.03^+^–5.8^+^, upper value censored) and duration of response (DOR) was 2.9 (2.8–3.1^+^) months. The most common G 3/4 toxicities included thrombocytopenia, neutropenia, febrile neutropenia and anemia in six, five, four and three patients, respectively. The combination warrants further studies with consideration to specific histological subgroups [75]. The combination of eribulin and cyclophosphamide was assessed in a 3 + 3 design phase Ib study in patients with taxane-resistant MBC. Eribulin was administered in two escalating doses on day 1 and 8, with cyclophosphamide 600 mg/m^2^ on day 1 every 21 days. Six patients were enrolled. No DLTs were observed. Patients received a median of 5.5 cycles (range 3–13), with three patients still on treatment at the time of presentation of the study. Due to the acceptable toxicity and promising activity, a phase II study in MBC is underway [69]. A phase Ib, dose-escalation study of eribulin, in combination with capecitabine, was conducted in patients with advanced solid refractory tumors. Eribulin by Schedule 1 (1.2, 1.6 or 2.0 mg/m^2^ on day 1) or Schedule 2 (0.7, 1.1 or 1.4 mg/m^2^ on days 1 and 8), in combination with twice-daily oral capecitabine (1000 mg/m^2^ oral BID, days 1–14 every 21 days), was administered. Thirty-four and 15 patients were recruited in Schedules 1 and 2, respectively. No unexpected toxicities were seen. The MTD for eribulin was 1.6 for Schedule 1 and 1.4 mg/m^2^ for Schedule 2, in combination with capecitabine 1000 mg/m^2^ twice-daily. Eribulin and capecitabine pharmacokinetics did not seem to interact. Schedule 2 MTD, due to high dose intensity, was selected for evaluation in a phase II breast cancer patients [70]. 

The effect of eribulin on cardiac repolarization was assessed in an open-label, single-arm, phase I study on patients with advanced solid tumors. Twenty-six patients were enrolled. No new or unexpected adverse events were reported. Eribulin demonstrated an acceptable safety profile with a minor QTc prolongation which is not expected to be of clinical concern [64]. However it is advised to avoid eribulin in congenital long QT syndrome [15]. Eribulin in combination with gemcitabine and cisplatin was evaluated as first-line therapy for locally advanced or metastatic bladder cancer in a phase Ib/II study. In the phase Ib part, three ascending doses of eribulin (on days 1 and 8 every 21 days) were administered to determine the MTD with standard doses of gemcitabine (1000 mg/m^2^, days 1 and 8 every 21 days) and cisplatin (70 mg/m^2^, day 1). Nine patients entered phase Ib. Eribulin was administered at 0.7 mg/m^2^ (*n* = 3) or 1.0 mg/m^2^ (*n* = 6), with standard dose of gemcitabine and cisplatin. One DLT of G 4 thrombocytopenia was observed at 1.0 mg/m^2^. The 1.4 mg/m^2^ dose was not explored due to high probability of developing severe hematologic DLTs. Thus, the MTD was neither achieved nor defined. The combination showed encouraging activity and manageable toxicities. The phase II part is currently ongoing, in which patients are to be randomized 1:1 to eribulin with gemcitabine and cisplatin, or gemcitabine and cisplatin alone [71]. A phase I, dose-escalation study of eribulin and S1 combination was conducted on MBC patients with prior treatment with both anthracyclines and taxanes. In Level 1, patients received S-1 (65 mg/m^2^, oral, days 1 to 14), and eribulin (1.1 mg/m^2^, i.v., day 1 and 8) in a 21-day cycle. In level 2, eribulin was increased to 1.4 mg/m^2^ and in level 3; S-1 was increased to 80 mg/m^2^. Twelve patients were enrolled and G3 hypokalemia as DLT was encountered at level 3. Eribulin pharmacokinetics was not affected by S1. At data cut-off seven patients were alive and one was on study. The ORR was 41.7% (95% CI: 8.9–74.4), median PFS was 7.6 months (95% CI: 1.3–NA) and the OS was not reached. The study warrants more investigation due to small size [72].

An open-label, multicenteric, randomized phase Ib/II study of eribulin administered in combination with pemetrexed *versus* pemetrexed alone as a second-line therapy in patients with advanced non-squamous NSCLC was recently conducted. In the phase Ib part dose escalation was carried in a 3 + 3 fashion with two ascending dosing arms. In arm one, eribulin (dosage 0.9, 1.4 or 2.0 mg/m^2^) was to be administered only on day 1 of a 21 day cycle. In the second arm, eribulin (dosage 0.7, 1.1 or 1.4 mg/m^2^) was to be administered on days 1 and 8 of a 21 day cycle. Both arms received the same dose of pemetrexed (500 mg/m^2^) on day 1 in combination with eribulin. In total, 15 patients were enrolled. Eribulin 0.9 mg/m^2^ in combination with pemetrexed on day 1 was defined as the MTD for treatment arm one and phase II study proceeded with this dose. MTD could not be defined for the second arm. Observed DLTs in arm one were febrile neutropenia (two patients), G 3 AST and ALT (1 patient), G 4 neutropenia, and G 4 thrombocytopenia (one patient). In the second arm, observed DLTs were G 4 transaminitis (one patient) and G 4 pneumonia (one patient) [73]. A Phase I trial was designed to determine the MTD of neoadjuvant eribulin mesylate, carboplatin, and trastuzumab (ECH) for operable HER2 positive breast cancer, with a planned follow-on Phase II component with pathological complete response as the primary endpoint. In this single-arm trial, eligible patients had operable stage IIA–IIIB HER2+ breast cancer. A starting dose of eribulin was 1.1 mg/m^2^, with escalation to dose level +1 at 1.4 mg/m^2^. Treatment was given for six three-week cycles with eribulin, days 1 and 8; carboplatin AUC 6, day 1; and trastuzumab 8 mg/kg loading dose day 1 cycle 1 and 6 mg/kg on day 1 in rest of the cycles. Overall 5/6 patients at 1.1 mg/m^2^ and 4/6 at 1.4 mg/m^2^ completed six cycles of ECH. Overall, 8 of 12 (67%) patients required PRBC transfusions (range 2–12 units) and two patients required platelet transfusions (range, 4–12 units). Eleven of 12 (92%) patients required dose-reduction of eribulin. The ECH regimen was associated with much higher levels of hematologic toxicities and transfusion requirements. The combination is not planned for further Phase II development in the HER2+ neoadjuvant setting [74].

### 5.2. Phase II Trials

#### 5.2.1. Breast Cancer

Table 3 provides a summary of four phase II trials of eribulin in MBC patients. The first phase II trial initially progressed on a 28 day schedule [48,76]. However, due to dosing difficulty on day 15, due to neutropenia, the treatment schedule was modified to days 1 and 8 of a 21-day cycle which proved to be a better toxicity profile [76,77]. The primary objective in all trials was to assess ORR. None of the trials showed any CR. Also no grade 4 neuropathy was seen. The results showed significant eribulin activity in different MBC settings and an acceptable safety profile [76,77,78,79].

**Table 3:** Phase II trials of eribulin in metastatic breast cancer patients.

	Vahdat *et al.* [76]	Cortes *et al.* [77]	Aogi *et al.* [78]	McIntyre *et al.* [79]
Protocol population	87	269	80	56
Patient criteria	prior therapy with at least an anthracycline and a taxane	2–5 prior chemo regimens including anthracycline, taxane and capecitabine (≥1 in metastatic or recurrent setting)	≤3 prior chemo regimens in metastatic setting including anthracycline and taxane	HER-2 neg, no prior chemo, biologic or investigational therapy in recurrent or metastatic setting
Median number of prior chemotherapy regimens (Range)	4 (1–11)	4 (2–5)	3 (1–5)	NA
ORR % (95% CI)	11.5 (5.7–20.1)	9.3 (6.1–13.4)	21.3 (12.9–31.8)	28.6 (17.3–42.2)
CBR % (95% CI)	17.2 (10.0–26.8)	17.1 (12.8–22.1)	27.5 (18.1–38.6)	51.8 (38.0–65.3)
Median DOR in months (Range)	5.6 (1.4–11.9)	4.1 (1.4^+^–8.5)	3.9 (1.0^+^–7.3^+^)	5.8
Median PFS in months (Range)	2.6 (0.03–14.9)	2.6 (0.03–13.1)	3.7 (0.3–14.8^+^)	6.8
Median OS in months (Range)	9 (0.5–27.1)	10.4 (0.6–19.9)	11.1 (1.0–25.9^+^)	UNK
Most common grade 3/4 toxicities (%)	Neutropenia (64), leukopenia (18), fatigue(5)	Neutropenia (54), leukopenia (14.1), fatigue (10)	Neutropenia (95.1), leukopenia (74.1), febrile neutropenia (13.6)	Neutropenia (50%), leukopenia (21%), and peripheral neuropathy (20%)
Grade 3 neuropathy (%) *	5	6.9	3.7	20

CBR—Clinical benefit rate (defined as CR + PR + SD > 6 months); CI—Confidence Interval; ^+^Censored observation, PFS—Progression-free survival, OS—Overall survival, PR-Partial response, DOR—Duration of response; 1–No complete response (CR) in all four trials; ORR—Overall objective response rate = [CR+PR/number of eligible patients]; NA—Not applicable; UNK—Unknown; * No grade 4 peripheral neuropathy in any trial.

A phase II, single-arm study evaluated the combination of eribulin and capecitabine for adjuvant treatment in post-menopausal ER+ early-stage breast cancer. In the study stage I-II, HER2 negative, ER+, female breast cancer patients received eribulin mesylate (1.4 mg/m^2^, i.v., day 1 and 8) and capecitabine (900 mg/m^2^, oral BID, days 1–14) on a 21 day cycle, for four cycles. Sixty-seven patients were enrolled, 64 patients were evaluable for feasibility and 59 completed four cycles of treatment. The study met its primary endpoint with a feasibility rate of 81% and average relative dose intensity (RDI) of 91%. Capecitabine dose adjustments (RDI—88%) were comparatively higher than that of eribulin (RDI—3%). As compared to eribulin, capecitabine had higher dose reductions (36% *vs.* 21%), missed doses (85% *vs.* 8%), and discontinuations due to adverse effects (16% *vs.* 10%). Most common G 3/4 toxicities leading to drug discontinuation were hand foot syndrome (8% patients), neutropenia (3%), neuropathy (2%), and gastrointestinal disorders (3%). Overall, 14 (21%) patients had an SAE. The adjuvant combination of eribulin with capecitabine is safe, with a majority of patients achieving full dosing regimen. An alternate schedule of capecitabine with seven weeks, on-and-off, with this regimen is being explored [80]. 

A phase II, randomized, open-label trial was conducted to compare the incidence and severity of neuropathy associated with eribulin with ixabepilone in MBC patients [81]. After 1:1 randomization, patients received eribulin (*n* = 51, 1.4 mg/m^2^, on days 1 and 8) or ixabepilone (*n* = 50, 40 mg/m^2^, on day 1) of a 21-day cycle. A median of 5.0 cycles of eribulin and 3.5 cycles of ixabepilone were administered. After controlling for pre-existing neuropathy (G 0 or 1) and number of prior chemotherapies (≤3, >3), the overall incidence of neuropathy and peripheral neuropathy between both treatments was not statistically significant (*p* = 0.1284 and *p* = 0.1632, respectively). With eribulin, neuropathy onset was later (35.9 weeks *vs.* 11.6 weeks) and resolved later (48 weeks *vs.* 10 weeks). Additionally, as compared to ixabepilone, fewer patients on eribulin discontinued treatment due to neuropathy (3.9% *vs.* 18.0%) or adverse events in general (11.8% *vs.* 32.0%). In intent to treat population, eribulin showed better activity as compared to ixabepilone in terms of ORR (15.4% *vs.* 5.8%), CBR (26.9% *vs.* 19.2%), DCR (67.3% *vs.* 55.8%), and median PFS (104 days *vs.* 95 days). The study was limited due to a small sample size and lacked the power to detect the observed magnitude of differences between the two treatment arms [81]. 

In a multicenter phase II study eribulin has been investigated in combination with ramucirumab, a recombinant human IgG1 monoclonal antibody directed against the vascular endothelial growth factor receptor 2. The primary goal was to identify if the combination would increase PFS in comparison to eribulin alone as third- to fifth-line therapy in patients with MBC. Patients were randomized in 1:1 to receive eribulin (1.4 mg/m^2^, days 1 and 8) or ramucirumab with eribulin (eribulin 1.4 mg/m^2^ on days 1 and 8; ramucirumab 10 mg/kg day 1) of 21 day cycle. Overall 141 patients were in the intent to treat population. Results are presented in Table 4. Addition of ramucirumab to eribulin did not improve PFS. The combination arm also had more toxicities (any G) of fatigue (64% *vs.* 57%), headache (39% *vs.* 15%), hypertension (13% *vs.* 1.5%), diarrhea (25% *vs.* 15%), and bleeding (18.8% *vs.* 4.6%), as compared to eribulin alone [82].

**Table 4:** Results of phase II study of ramucirumab plus eribulin *versus* eribulin alone in advanced metastatic breast cancer patients [82].

	Ramucirumab with Eribulin (*n* = 71)	Eribulin (*n* = 70)
Median progression free survival (months)	4.4	4.1 (HR = 0.8: 95% CI: 0.6–1.2; *p* = 0.4)
median overall survival (months)	13.5	11.5 (HR = 0.8: 95% CI: 0.5–1.3; *p* = 0.4)
Objective response rate	20%	24%
Median duration of response (months)	5.5	3.0
Relative mean dose intensity	95.3% for ramucirumab and 80.7% for eribulin	79.0%

In a multicenter, phase II, single-arm study eribulin was administered with trastuzumab as a first-line therapy in patients with recurrent or metastatic HER2+ breast cancer. Eribulin (1.4 mg/m^2^) on days 1 and 8 of 21-day cycle was administered with an initial trastuzumab dose of 8 mg/kg on day 1, followed by 6 mg/kg on day 1 of each subsequent cycle. Overall, 52 patients were enrolled. A median of 10.0 cycles of eribulin and 11.0 cycles of trastuzumab were administered. Thirty-seven patients (71.2%, 95% CI: 56.9–82.9) experienced ORR (primary end-point) with a median DOR of 11.1 months (95% CI: 6.7–17.8), and a median PFS of 11.6 months (95% CI: 9.1–13.9). Kaplan-Meier PFS estimates at 3-, 6-, 9-, and 12-month were 96%, 82%, 67%, and 49%, respectively. The most common G3/4 treatment-emergent adverse events were neutropenia (38.5%), peripheral neuropathy (26.9%; all G 3), fatigue (7.7%), and febrile neutropenia (7.7%). One death due to chronic heart failure was considered to be possibly related to study drug. The combination seems interesting due to its high ORR and prolonged PFS and predictable safety profile [83]. 

In a phase II randomized, neo-adjuvant clinical trial, weekly paclitaxel or eribulin was followed by doxorubicin and cyclophosphamide in women with locally advanced HER2-negative breast cancer. The primary endpoint was pathologic complete response in the breast and nodes. A total of 50 patients were accrued. They were randomly assigned in 2:1 to receive either eribulin (*n* = 31, 1.4 mg/m^2^ day 1 and day 8) every three weeks for four cycles, or weekly paclitaxel (*n* = 19, 80 mg/m^2^) for 12 treatments. Both groups received doxorubicin and cyclophosphamide (60 mg/m^2^ and 600 mg/m^2^ respectively) every three weeks for four cycles before surgery. Significant G 3 toxicities included diarrhea, febrile neutropenia, mucositis, and thromboembolic events (2%, one patient each). Of available MRI results weekly paclitaxel showed better response of 83% (15/18 pts, 14 PR and 1 CR) as compared to 57% (15/26 patients, 14 PR and 1 CR) with eribulin by investigator and/or central review. By central review weekly paclitaxel had 50% response as compared to 45% with eribulin. Available data for primary endpoint of pathological complete response also favors weekly paclitaxel (7/19, 37%), as compared to eribulin (6/31, 19%). Final results are awaited [84]. 

Eribulin is being tested at a lower dose metronomic schedule in an open-label, multi-center, phase II study in patients with MBC. Patients will receive eribulin 0.9 mg/m^2^ intravenously over 2 to 5 min on days 1, 8, and 15 of a four week cycle. Eligibility criteria include patients with MBC with disease progression following 1–6 prior regimens with prior exposure to a taxane and measurable disease. The primary objective is to assess PFS and secondary objectives include a decrease in the frequency of alopecia to less than 50%, a decrease in incidence of G 3/4 neutropenia to less than 30% and a decrease in incidence of sensory neuropathy (all grades) to less than 25%. A tertiary objective of the study is to assess the role of apoptotic circulating endothelial cells and circulating endothelial cell precursors in predicting early response to treatment. As per available data 12 of 60 patients have been enrolled [85].

#### 5.2.2. Non-Small Cell Lung Cancer

A single-arm, phase II study of eribulin was conducted in patients with advanced NSCLC that have progressed after platinum-based doublet chemotherapy. Eribulin mesylate (1.4 mg/m^2^) was administered on days 1, 8, and 15 of a 28-day cycle which was later changed to 21 day schedule due to difficulty in administering treatment on day 15, as in above phase II breast trials [76]. Patients were enrolled in two cohorts based on prior taxane exposure. A total of 103 patients were treated, of which 83 were with prior taxane therapy and 20 were taxane naïve. A median number of three (range 1–15) cycles were administered. The primary efficacy endpoint, ORR (all PRs) was 9.7% (95% CI: 4.8–17.1), with 10.8% (95% CI: 5.1–19.6) in taxane pre-treated, and 5% (95% CI: 0.1–24.9) in taxane naïve patients. The DCR (CR + PR + SD) was 55.3% (95% CI: 45.2–65.1), median PFS was 3.4 months (95% CI: 2.4–3.6), median DOR was 5.8 months (range, 1.6 to 9.6+ months), and median OS was 9.4 months. The most common G 3/4 toxicities were neutropenia (49%), fatigue (11%) and leucopenia (6%). Eribulin showed activity in patients with NSCLC with prior taxane therapy and was tolerated as second or later line [86].

In another phase II trial of eribulin in NSCLC patients with prior treatment with platinum-based therapy and a taxane and up to two cytotoxic chemotherapy regimens for either metastatic disease or as adjuvant therapy were recruited. Eribulin was administered as 1.4 mg/m^2^ on day 1 and 8 of a 21-day schedule. Patients were classified in two strata based on taxane-sensitivity. The primary endpoint was ORR. In the taxane sensitive stratum (progression >90 days after taxane), 45 patients were enrolled. A median number of four cycles (range 1–23) were administered. Three (7%) patients had PR, median PFS was 2.9 months (95% CI: 2.5–4.8) and median OS was 12.6 (95% CI: 9.9–17.5). In the taxane-resistant stratum (progression during or <90 days after taxane) 21 patients were enrolled. A median number of two cycles (range 1–8) were administered. No response was seen, median PFS was 1.2 months (95% CI: 1.1–2.9) and median OS was 8.9 months (95% CI: 5–15.4). Common G 3/4 drug related toxicities neutropenia (55%), leucopenia (29%) and fatigue (9%). The ORR was 5% with median DOR of 7.8 months. Eribulin showed encouraging activity in taxane sensitivity NSCLC [87].

The phase II part of the study of eribulin with pemetrexed *versus* pemetrexed alone, as mentioned above [73], was a two-arm design conducted on patients with nonsquamous NSCLC with locally advanced or metastatic disease. All patients received study treatment as second-line therapy, although one additional cytotoxic regimen was allowed for neoadjuvant, adjuvant, or neoadjuvant with adjuvant therapy. Patients were randomized in 1:1 to receive either eribulin (0.9 mg/m^2^) with pemetrexed (500 mg/m^2^) (as previously defined in the phase Ib part of the study) or pemetrexed (500 mg/m^2^) alone on day 1 of every 21-day cycle. The combination of eribulin was safe and tolerated well, but did not show a therapeutic benefit over pemetrexed alone. The results are summarized in Table 5 [73].

**Table 5:** Phase II Study of Eribulin Mesylate Administered in Combination With Pemetrexed *versus* Pemetrexed Alone [73].

	Eribulin with Pemetrexed	Pemetrexed
Patients enrolled/treated/modified intent to treat	42/41/39	41/39/39
Imputed median progression free survival (weeks)	21.4 (*n* = 26; 95% CI: 12.7–39.6)	23.4 (*n* = 29; 95% CI: 17.1–29.9), HR 1.0 (95% CI: 0.6–1.7)
Imputed median time to progression (weeks)	21.4 (*n* = 24; 95% CI: 12.7–39.6)	23.4 (*n* = 27; 95% CI: 17.1–29.9), HR 1.1 (95% CI: 0.6–1.9)
Median overall survival (weeks)	59.1 (*n* = 14; 95% CI: 27.7–not reached)	(*n* = 15; 95% CI: 29.4–not reached), HR 1.0 (95% CI: 0.5–2.0).
Overall response (all partial)	Eight patients (20.5%; 95% CI: 7.8%–33.2%)	Six patients (15.4%; 95% CI: 4.1%–26.7%)
Patients experiencing progressive disease or death at 12 weeks	15 (38.5%; 95% CI: 23.2%–53.7%)	12 (30.8%; 95% CI: 16.3%–45.3%).
Most common grade ≥3 adverse events	Neutropenia (17%), anemia (10%), and increased ALT (10%)	Neutropenia (18%), increased ALT (18%), increased AST (15%)

Eribulin with erlotinib was investigated in two intercalated combinations in an open-label, randomized, phase II study on patients with advanced NSCLC previously treated with platinum-based chemotherapies. Patients were randomized to eribulin (2.0 mg/m^2^) on day 1 with erlotinib (150 mg) on days 2–16 (21-day cycle) or eribulin (1.4 mg/m^2^) on days 1 and 8 with erlotinib (150 mg) on days 15–28 (28-day cycle). The primary end point was an objective response rate (ORR). Results are summarized in Table 6. Most common G ≥ 3 AEs for the 21 and 28 day regimens included neutropenia (56% *versus* 48%), asthenia/fatigue (13% *versus* 12%), and dyspnea (10% for both). The 28 day regimen seemed more tolerable, as 21 day regimen seemed to have a higher incidence of febrile neutropenia (17% *versus* 5%), AE related dose-reductions (40% *versus* 27%), SAEs (60% *versus* 45%), and AEs leading to study drug withdrawal (24% *versus* 10%). Eribulin pharmacokinetics were not affected by erlotinib. Both regimens seemed to be similarly efficacious, however, the combination did not seem to improve treatment outcomes in advanced NSCLC patients without biomarker selection [88].

**Table 6:** Phase II study of two intercalated combinations of eribulin and erlotinib in patients with previously treated NSCLC [88].

	21 Day Regimen	28 Day Regimen
Intent to treat population/Evaluable for response	63/62	60/58
Median number of cycles (Range)	Three (1–44)	Four (1–33)
ORR	13% (95% CI: 6%–24%)	17% (95% CI: 8%–29%)
Disease control rates	48% (95% CI: 35%–61%)	63% (95% CI: 50%–75%)
Median DOR (months)	9.4 (95% CI: 2.7–censored)	9.7 (95% CI: 5.6–censored)
Median PFS (months)	3.5 (95% CI: 1.9–4.7)	3.8 (95% CI: 3.3–5.5)
OS (months)	7.6 (95% CI: 6.3–11.0)	8.5 (95% CI: 6.2–13.1)

PFS—Progression-free survival; OS—Overall survival; DOR—Duration of response; ORR—Overall objective response rate = [CR + PR/number of eligible patients]; CI—Confidence Interval.

#### 5.2.3. Prostate Cancer

In the first open-label, two-stage design, single-arm, phase II study in metastatic castration-resistant prostate cancer (CRPC) patients, eribulin was administered as 1.4 mg/m^2^ on days 1 and 8 of a 21-day cycle. Patients were evaluated in two separate strata based on prior taxane exposure. The primary efficacy endpoint was a prostate-specific antigen (PSA) response rate, based on Bubley criteria. Results are summarized in Table 7. Eribulin showed some activity in metastatic CRPC patients with taxane-naïve disease [89].

In a second multicenter trial, 119 metastatic CRPC patients received eribulin with the same treatment schedule as the above trial. The median number of treatment cycles was four (range 1–20^+^). In this non-comparative study patients were stratified to either a chemo-naïve arm, prior-taxane only arm, or two prior cytotoxic chemotherapy arms. The trial was powered to detect a 50% PSA reduction using Consensus Criteria in at least 40% *vs.* 20% (90% power, one-sided α = 0.10) for the chemo-naïve stratum and 25% *vs.* 10% (power 87%, one-sided α = 0.10) for the taxane and two prior cytotoxic chemotherapy strata. The chemo-naïve arm had 41 patients, 24% had ≥50% PSA response, and 8% (of 26 patients with measurable disease) showed response. Median duration of PSA response was 7.1 months and median OS (in months) was not reached. The prior taxane arm had 51 patients, 10% had ≥50% PSA response, and 3% (of 38 patients with measurable disease) showed response. Median duration of PSA response was 3.6 months and median OS was 11.4 months. The “two prior cytotoxic chemotherapy regimens” arm had 51 patients, 4% had ≥50% PSA response, and 8% (of 13 patients with measurable disease) showed response. Median duration of PSA response cannot be evaluated and median OS was 13.7 months. Important treatment related G 3/4 toxicities in taxane-naïve, prior taxane, and two prior chemotherapies strata were neutropenia (52%, 50%, 68%), leukopenia (33%, 44%, 52%), fatigue (17%, 8%, 12%), and sensory neuropathy (14%, 16%, 4%), respectively. The results, though, demonstrated some activity of eribulin in metastatic CRPC with taxane-naïve disease; the ORR was not sufficient to warrant further studies in CRPC [90].

**Table 7:** Comparison of metastatic castration-resistant prostate cancer patients stratified by prior taxane therapy treated with eribulin [89].

	Taxane-Naïve	Taxane-Pretreated
Safety population	58	50
Efficacy population	58	47
Patients with measurable disease (%)	33 (56.9)	29 (61.7)
Median eribulin cycles (range)	4.0 (1–47)	3.0 (1–16)
PSA response (≥50% decline) (%, 95% CI)	22.4% (12.5–35.3)	8.5% (2.4–20.4)
No. of Patients with measurable disease (%)	33 (56.9)	29 (61.7)
No. of patients with PR (%)	5 (15.2)	0
No. of patients with SD (≥12 weeks) (%)	25 (75.8)	20 (69.0)
ORR (95% CI)	15.2 (5.1–31.9)	0
Median OS in months (range)	20.8 (2.2^+^–32.4^+^)	15.0 (1.0^+^–32.4^+^)
Median PFS in months (range)	2.1 (0.03^+^–32.2^+^)	1.9 (0.03^+^–9.9)
Treatment related G 3/4 toxicities	Neutropenia (22.4%), leucopenia (8.6%), fatigue (6.9%)	Neutropenia (40.0%), leucopenia (16%, respectively), fatigue (8.0%)

PFS—Progression-free survival; OS—Overall survival; PR—Partial response; ORR—Overall objective response rate = [CR + PR/number of eligible patients]; CI—Confidence Interval; ^+^ censored observation.

#### 5.2.4. Ovarian Cancer

In a phase II study epithelial ovarian, fallopian tube or peritoneal cancer patients with measurable disease and ≤2 prior cytotoxic regimens received eribulin (1.4 mg/m^2^) on days 1 and 8 of every 21 days cycle. Patients were stratified into platinum-resistant (progression-free interval from last platinum-based therapy <6 months) and platinum-sensitive (progression-free interval from last platinum-based therapy ≥6 months) cohorts. The primary end-point was ORR. The results are presented in Table 8. No CR was observed in the study. Eribulin showed activity in platinum-sensitive (ORR 19%) but was inactive in platinum-resistant (ORR 5.5%) recurrent ovarian cancer [91].

**Table 8:** Results of platinum-resistant and platinum-sensitive ovarian cancer patient cohorts treated with eribulin [91].

	Platinum Resistant Cohort	Platinum Sensitive Cohort
Patients enrolled/evaluable	37/36	37/37
Median age in years (range)	61 (38–80)	60 (45–77)
Median no. of cycles delivered (range)	2 (1–10)	6 (1–51)
Partial response (%)	2 (5.5%)	7 (19%)
Stable disease (%)	16 (44%)	21 (57%)
Median PFS (months) (95% CI)	1.8 (1.4–2.8)	4.1(2.8–5.8)
Median OS (months) (95% CI)	18 (11–25)	26 (21–38)
Treatment related G3/4 toxicities (>10%)	Neutropenia (42%) and leucopenia (33%)	Neutropenia (54%) and leucopenia (30%)

PFS—Progression-free survival; OS—Overall survival; CI—Confidence Interval.

A phase II study to evaluate the effect of weekly administration of bevacizumab (2 mg/kg) with eribulin (1 mg/m^2^) and oxaliplatin (30 mg/m^2^) in patients with platinum-resistant and refractory ovarian carcinoma was performed. The study was based on Simon's two-stage design, in which 15 patients were to be accrued in the first stage and, if there were no response, the study would stop. Otherwise, eight more patients will be accrued. In the first stage four responses of 15 patients were observed and a total of 23 patients were analyzed. No treatment discontinuation was due to toxicities. The median number of prior regimens was four (range 2–8). Overall 2 (9%) patients had CR, three (13%) had PR and nine (39%) had a SD. The response rate was 17%. Median PFS was three months (range: 1–8^+^). Hematological G ≥ 3 toxicities were observed in four patients (17%) and G ≥ 3 Hypoalbuminemia and edema in one patient (8%), respectively. The weekly treatment with bevacizumab, eribulin, and oxaliplatin showed significant activity with acceptable toxicities. The study warrants further investigations [92]. 

#### 5.2.5. Sarcoma

Efficacy and safety of eribulin in soft tissue sarcoma was assessed in a non-randomized phase II study. Eligibility criteria included patients with intermediate or high G, histologically proven, advanced or metastatic soft-tissue sarcomas with up to two single agents or one combination for advanced disease. Eribulin, (1.4 mg/m^2^) was given on days 1 and 8 every three weeks. Patients were enrolled in four independent strata, namely leiomyosarcoma, adipocytic, synovial, and other defined soft tissue sarcomas. The primary end point was the PFS at 12 weeks. A Simon two-stage design was employed (P1: 40%; P0: 20%; α = β = 0.1) per stratum. A median of four cycles (range of 1 to 43^+^) per patient were given. Results are summarized in Table 9. Important treatment-related toxicities (all G) included anemia (88%), leucopenia (84% patients), and neutropenia (80%). The results showed eribulin activity in leiomyosarcoma and adipocytic sarcoma subgroups, as the PFS at 12 weeks reached predefined statistical targets with manageable toxicities. A randomized phase III trial in patients with leiomyosarcoma and adipocytic sarcoma is ongoing [93].

**Table 9:** Results of eribulin activity in soft-tissue sarcoma subtypes [93].

	Leiomyosarcoma	Adipocytic Sarcoma	Synovial Sarcoma	Other Soft Tissue Sarcoma
Total/evaluable patients	40/38	37/32	19/19	32/26
Median age in years (range)	60.1 (27.9–81.4)	59.2 (32.7–75.2)	42.3 (20.8–73.9)	55.9 (18.0–83.3)
No. of patients progression free at 12 weeks (%, 95% CI)	12 (31.6, 17.6–48.7)	15 (46.9, 29.1–65.3)	4 (21.1, 6.1–45.6)	5 (19.2, 6.6–39.4)
Complete response (%)	0	1 (3%)	0	0
Partial response (%)	2 (5%)	0	1 (5%)	1 (4%)
Stable disease (%)	20 (53%)	18 (56%)	8 (42%)	11 (42%)
Median PFS in months (95% CI)	2.9 (2.4–4.6)	2.6 (1.7–6.2)	2.6 (2.3–4.3)	2.1 (1.4–2.9)
6-month OS (%) (95% CI)	86.8 (71.2–94.3)	74.6 (55.5–86.4)	71.1 (43.7–86.8)	52.9 (31.2–70.7)

PFS—Progression-free survival; OS—Overall survival; CI—Confidence Interval.

Eribulin was also evaluated in another open-label, multicenteric, single-arm, phase II study in Japanese patients with advanced soft tissue sarcomas who had received at least one standard chemotherapy. Overall 51 patients with high G (*n* = 38, 74.5%) or intermediate G (*n* = 13, 25.5%) soft tissue sarcoma were treated. Eribulin mesylate (1.4 mg/m^2^) on day 1 and 8 of 21-day cycle was given. Patients were divided in two independent strata, namely (1) adipocytic (*n* = 16) or leiomyosarcoma (*n* = 19), and (2) other histological types (*n* = 16). The primary endpoint was the progression-free rate at 12 weeks. All patients received anthracycline and 36 (70.6%) patients received ifosfamide as prior chemotherapy. In adipocytic/leiomyosarcoma strata, progression-free rate at 12 weeks was 60.0% (21/35 pts; 95% CI: 42.1%–76.1%), median PFS was 5.5 (95% CI: 2.8–8.2) months, 1 year OS was 60% and 80% had SD. In other histological types strata progression-free rate at 12 weeks was 31.3% (5/16; 95% CI:, 11.0–58.7), median PFS was 2.0 (95% CI: 1.2–4.1) months, 1 year OS was 50% and 50% had SD. Overall study population had a progression-free rate at 12 weeks of 51.0% (26/51 patients; 95% CI: 36.6–65.2), median PFS was 4.1 (95% CI: 2.6–5.6) months, 1 year OS was 56.9% and 70.6% had SD. No response was seen in the study. The most common treatment-related G ≥ 3 toxicities were neutropenia (86.3%), leukopenia (74.5%), lymphopenia (31.4%), anaemia (11.8%), and febrile neutropenia (7.8%). Eribulin showed some activity in advanced pre-treated soft tissue sarcoma patients with predictable toxicities [94]. 

#### 5.2.6. Pancreatic Cancer

Eribuin was evaluated as a second-line therapy in an open-label, multi-centric, single-arm, phase II study in pancreatic cancer patients. Eligibility criteria included measurable disease and prior gemcitabine therapy. The study employed Simon two-stage design, with ORR as the primary endpoint. Eribulin mesylate (1.4 mg/m^2^) was administered on days 1 and 8 of 21 day cycle. Fifteen patients were enrolled, 14 received treatment and 12 were evaluable for response. A median number of two cycles (range 1–16) were administered. The study was closed at the end of stage 1 due to no response. Stable disease as best response was seen in five (42%) patients of which three had SD for 12 cycles or more (range 12–16 cycles). Median time to progression was 1.4 months (95% CI: 1.2–8.5), and median OS was 6.1 months (95% CI: 1.4–20.8). Drug related G 3/4 toxicities included neutropenia (29%), leucopenia (21%), and fatigue (14%). Though no response was seen, due to long term disease control and manageable toxicities further studies of eribulin in pancreatic cancer may be warranted [95].

#### 5.2.7. Urothelial Tract Cancer (UC) and Renal Insufficiency (UCD)

After encouraging results of eribulin in phase I renal dysfunction and advanced urothelial cancer [62], a subsequent phase II part was conducted which included a two-stage design (requiring ≥2 responses in 21 patients to proceed to a total of 41 patients). Initially, only patients without renal insufficiency were to be enrolled until the phase II MTD was proved safe. Thereafter, accrual of patients with renal insufficiency was to begin. In the first part, patients with normal creatinine or calculated CrCl ≥60 mL/min and histologically or cytologically confirmed urothelial cancer without prior cytotoxic therapy for advanced disease were accrued. Neo and adjuvant therapies were allowed. Eribulin mesylate (1.4 mg/m^2^) was administered on day 1 and 8 of 21 day cycle. The primary objective was ORR evaluation. Forty patients entered the trial which included transitional (35), adenocarcinoma (three), squamous (one), and small cell (one) cancer. The percentage of patients in Bajorin risk groups 0, 1 and 2 were 30%, 57% and 13% respectively. Of the 37 evaluable patients the ORR was 38% (95% CI: 23–54, 1 CR and 14 PR) including 13 responses in transitional cell cancer patients. Overall 72.5% patients received prior neo/adjuvant chemotherapy and among them a response rate of 34% was observed. At median follow-up of 19.8 months, the median PFS was 3.9 months (95% CI: 2.7–5.3) and the median OS was 9.4 months (95% CI; 6.7–11.9). A significant correlation between PFS and Bajorin risk group (*p* = 0.028 for trend) was observed. Important G 3/4 drug related toxicities included neutropenia (20 patients), hyponatremia (four patients), hyperglycemia (three patients), sensory neuropathy (one patient), and leg fatigue with aching (one patient). The results demonstrated significant activity of eribulin in urothelial cancer to warrant further investigations. Per last reports in patients with CrCl <40 L/min, 1.4 mg/m^2^ dose was tolerable [96].

#### 5.2.8. Squamous Cell Carcinoma of the Head and Neck

Eribulin activity in patients with metastatic or recurrent squamous cell carcinoma of the head and neck (SCCHN) was evaluated in a multi-institutional phase II trial. The main objective was the assessment of response rate. Eligibility criteria included no prior chemotherapy for recurrent or newly diagnosed metastatic disease. Only one induction or adjuvant therapy was allowed. Forty patients were enrolled of which 33 (83%) had metastatic disease. Primary tumor sites included oropharynx (38%), lip/oral cavity (30%), larynx (15%), hypopharynx (10%), other/unknown (5%) and nasopharynx (3%). Eribulin was administered at 1.4 mg/m^2^ on days 1 and 8 of every 21-day cycle. Common G 3/4 toxicities included lymphopenia (15%), leucocytopenia (13%), neutropenia (10%), hyponatremia, fatigue, diarrhea, and dyspnea (5% each). One death due to treatment-related pulmonary hemorrhage was observed. A response rate of 5% (95% CI: 1%–17%), which included 2 PRs was observed. The median PFS was 3 months (95% CI: 1–3 months) and median OS was 7 months (95% CI: 5–10 months). Thus, though well-tolerated, eribulin did not show significant activity in this setting [97].

### 5.3. Phase III Studies

#### 5.3.1. Breast Cancer

To date, two randomized, phase III trials have been conducted with eribulin. The first of these trials, “EMBRACE” (Eisai Metastatic Breast Cancer Study Assessing Physician’s Choice Versus E7389; NCT00388726; Study 305), was a global, open-label, multicenter (137), randomized, controlled, parallel-group study which compared OS with eribulin to real-life choices. Inclusion criteria included locally-recurrent or MBC patients previously treated with 2–5 prior chemotherapies (≥2 for advanced disease), including an anthracycline and a taxane. Patients were randomized in 2:1 to eribulin (1.4 mg/m^2^, days 1 and 8 of a 21-day cycle) or treatment of the physician’s choice (TPC; defined as any approved single-agent chemotherapy or hormonal or biological treatment, radiotherapy, or symptomatic treatment alone). The primary endpoint was OS. In all, 762 (508 eribulin, 254 TPC) patients with a median number of four prior chemotherapy regimens (range 1–7) entered the study. Approximately 73% of them had received prior capecitabine. Results are summarized in Table 10. The study met its primary objective and showed a significant improvement in OS of 2.5 months with eribulin. Among secondary end-points ORR reached, but PFS did not reach, statistical significance on independent review. However, PFS was significant on investigator review [eribulin 3.6 months *vs.* TPC 2.2 months, hazard ratio (HR) 0.76, 95% CI: 0.64–0.90; *p* = 0.002]. This might be due to more patients being censored in independent review as compared to investigator review (241 *vs.* 127). Though eribulin showed a significant response rate (12% with eribulin *vs.* 5% with TPC; *p* = 0.002), it was on the lower side [14].

Another phase III open-label, randomized, multicenter, controlled, parallel-group study (NCT00337103; Study 301) was conducted to compare eribulin with capecitabine in patients with locally-advanced or MBC who had a prior therapy with an anthracycline and a taxane. Inclusion criteria included up to three prior chemotherapy regimens and up to two prior chemotherapy regimens for advanced and/or MBC. Patients were randomized 1:1 to eribulin mesylate (1.4 mg/m^2^ i.v., days 1 and 8 of a 21 day cycle) or capecitabine (1.25 g/m^2^ orally BID, days 1 to 14 of a 21 day cycle). In contrast to the EMBRACE study, eribulin was not superior to capecitabine with regard to either OS or PFS. Average global health status and overall quality of life scores improved with time in both arms and no significant difference was observed between the groups. Results are summarized in Table 9. Hematological side effects and peripheral neuropathy were more common with eribulin, whereas gastrointestinal side effects like nausea, vomiting, and diarrhea were more common with capecitabine [16]. 

**Table 10:** Summary of results of EMBRACE trial [14] and Phase III study of eribulin mesylate *versus* capecitabine [16] as assessed by independent review.

	EMBRACE Trial		Eribulin *vs* Capecitabine	
	Eribulin	TPC	Eribulin	Capecitabine
No. of patients randomized/treated	508/503	254/247	554/544	548/546
Age (range)	55.0 (28–85)	56.0 (27–81)	54.0 (24–80)	53.0 (26–80)
Median duration of treatment, months (range)	3.9 (0.7–16.3)	2.1 (0.03–21.2) for chemotherapy (*n* = 238), and 1.0 month (0.8–6.2) for hormone therapy (*n* = 9).	4.1 (0.7–45.1)	3.9 (0.7–47.4)
Median PFS, months (95% CI)	3.7 (3.3–3.9)	2.2 (2.1–3.4) (HR 0.87; 95% CI: 0.71–1.05; *p* = 0.137)	4.1 (3.5–4.3)	4.2 (3.9–4.8) (HR 1.08; 95% CI: 0.93–1.25; *p* = 0.30)
Number of patients with complete response (%)	3 (1)	0	1 (0.2)	0
Number of patients with partial response (%)	54 (12)	10 (5)	60 (10.8)	63 (11.5)
Number of patients with stable disease (%)	208 (44)	96 (45)	313 (56.5)	303 (55.3)
ORR % (95% CI)	12 (9.4–15.5)	5 (2.3–8.4; *p* = 0.002)	11.0 (8.5–13.9)	11.5 (8.9–14.5; *p* = 0.85)
CBR % (95% CI)	23% (18.9–26.7)	17% (12.1–22.5)	26.2 (22.6–30.0)	26.8 (23.2–30.7; *p* =0.84)
Median DOR (months) (95% CI)	4.2 (3.8–5.0)	6.7 (6.7–7.0; *p* = 0.159)	6.5 (4.9–6.0)	10.8 (6.8–17.8; *p* =0.01)
Median OS (months) (95% CI)	13.1 (11.8–14.3)	10.6 (9.3–12.5) HR 0.81 (95% CI: 0.66–0.99; *p* = 0.041)	15.9 (15.2–17.6)	14.5 (13.1–16.0) (HR 0.88; 95% CI: 0.77–1.00; *p* = 0.056)
Most common adverse events (all grades)	Asthenia/fatigue (54%), neutropenia (52%), alopecia (45%), peripheral neuropathy (35%)	Asthenia/fatigue (40%), neutropenia (30%), nausea (28%), anemia (23%)	Neutropenia (54.2%), alopecia (34.6%), leukopenia (31.4%), peripheral neuropathy (27.4%)	Hand-foot syndrome (45.1%), diarrhea (28.8%), nausea (24.4%), anemia (17.6%)
Adverse events leading to treatment discontinuation (%)	13	15	7.9	10.4
Serious adverse events (%)	25	26	17.5	21.1

CBR—Clinical benefit rate (defined as CR + PR + SD > 6 months); CI—Confidence Interval; PFS—Progression-free survival; OS—Overall Survival; DOR—Duration of response; ORR—Overall objective response rate = [CR + PR/number of eligible patients].

In a pooled analysis of both phase III studies, 1062 patients were found to be randomized to eribulin and 802 patients to control. Eribulin showed a significantly increased median OS (15.2 *vs*. 12.8 months, HR 0.85; 95% CI: 0.77, 0.95; *p* = 0.003) and median PFS (4 *vs*. 3.4 months, HR 0.90; 95% CI: 0.81, 0.997; *p* = 0.046) as compared to controls in the intent-to-treat population [98]. Eribulin showed an increased OS and PFS across all patient subgroups. In particular, eribulin showed a significant benefit in median OS in patients with HER2-negative disease (15.2 *vs*. 12.3 months; HR 0.82; *p* = 0.002) and triple negative disease (12.9 *vs*. 8.2 months; HR 0.74; *p* = 0.006) as compared to the control group. On interaction analysis, a distinct OS benefit with eribulin was seen in patients with more than two organ involvement (heavy tumor burden). In most subgroup analyses, the magnitude of benefit with eribulin was small and interaction analysis did not show that the benefits were specific to these subgroups. Eribulin showed a greater influence over OS as compared to PFS reasons for which are unclear. Additionally, it needs to be noted it was an unplanned analysis at the request of the European Medicines Agency and both studies involved different patient cohorts with regards to extent of prior chemotherapy [98].

To analyze effect of eribulin treatment on older patients, data from two single-arm, phase II studies [76,77] and the EMBRACE trial [14] were pooled. Overall, 827 patients with MBC were included in the analyses. A similar OS, PFS, ORR, CBR, and toxicity profile was seen in older (≥70 years) patients with MBC as compared to younger patients on treatment with eribulin (Table 11). Asthenia or fatigue (70.9% *vs*. 54.9%), peripheral edema (19% *vs.* 5.5%), and dizziness (12.7% *vs*. 5.9%) were more common in older patients (≥70 years), whereas nausea (22.8% *vs*. 40.7%) and vomiting (11.4% *vs.* 23.7%) were more common in youngest cohort (<50 years). Adverse events leading to dose reduction, delay or discontinuation increased slightly with age, though on analysis of cohorts no significant difference in the odds ratio for occurrence of adverse events leading to dose reductions, delays or withdrawal was observed. However, real-world application of this analysis is limited by the strict eligibility criteria for participation in clinical trials [99]. 

**Table 11:** Analysis of eribulin monotherapy from pooled patient cohorts by age from two phase II studies and EMBRACE trial [99].

	<50 Years	50–59 Years	60–69 Years	≥70 Years
ITT patients/ Evaluable patients	253/234	289/262	206/195	79/75
Median OS (months) in ITT	11.8	12.3	11.7	12.5
Median PFS (months)	3.5	2.9	3.8	4.0
ORR (%)	12.7	12.5	6.3	10.1
CBR (%)	20.2	20.8	20.4	21.5
Common adverse events (all grades) (%)	Asthenia/fatigue (54.9), neutropenia (49.8), alopecia (47.4)	Asthenia/fatigue (59.2), neutropenia (56.7), alopecia (51.2)	Asthenia/fatigue (62.6), neutropenia (59.2), alopecia (52.4)	Asthenia/fatigue (70.9), neutropenia (57), alopecia (51.9)
Common grade 3/4 adverse events (%)	Neutropenia (43.9), leucopenia (12.6), asthenia/fatigue (6.7)	Neutropenia (50.2), leucopenia (14.2), asthenia/fatigue (9.0)	Neutropenia (52.9), leucopenia (16.0), asthenia/fatigue (11.7)	Neutropenia (49.4), asthenia/fatigue (16.5), leucopenia (12.7)

ITT—Intent to treat; CBR—Clinical benefit rate (defined as CR + PR + SD > 6 months); PFS—Progression-free survival; OS—Overall survival; ORR—Overall objective response rate = [CR + PR/number of eligible patients].

#### 5.3.2. Lung Cancer

An open-label, parallel-group, phase III study was conducted to compare eribulin with TPC in patients with advanced NSCLC. Patients with advanced NSCLC and disease progression following ≥2 prior regimens for advanced disease (including platinum-based therapy) were randomized 1:1 to receive eribulin mesylate (270 patients,1.4 mg/m^2^ on days 1 and 8, every 21 days) or TPC (270 patients, 21-day cycles of vinorelbine, gemcitabine, pemetrexed [nonsquamous only] or docetaxel). Overall, 55.0% of patients had received ≥3 prior chemotherapy regimens, 33.3% of patients were aged >65 years, 61.5% were male and 20.9% had squamous histology. The primary endpoint was OS and secondary endpoints included PFS, ORR and safety and tolerability. Both eribulin and TPC arms had a median OS of 9.5 months (HR, 1.16 [95% CI: 0.95, 1.41]; *p* = 0.134). Median PFS with eribulin was 3.0 months and with TPC was 2.8 months (HR, 1.09 [95% CI: 0.90, 1.32]; *p* = 0.395). The ORR with eribulin was 12.2% and with TPC was 15.2%. Therefore, eribulin did not seem to improve OS or PFS as compared to TPC in patients with advanced NSCLC. The most frequent G 3/4 AEs with eribulin were neutropenia (28.6%), decreased neutrophil count (21.2%), and decreased WBC count (13.4%). Overall, 35.7% patients had serious adverse events compared to 32.1% with TPC [100].

## 6. Conclusions and Future Directions

Eribulin has a unique mechanism of action and has shown activity in a wide range to cancers. It has shown significant improvement in OS in heavily-pretreated refractory breast cancer patients in the EMBRACE trial as compared to TPC. Currently, two phase III trials are comparing eribulin in recurrent or MBC with vinorelbine and paclitaxel [17]. Another phase III study is comparing the efficacy and safety of eribulin with dacarbazine in patients with soft tissue sarcoma [17]. It has also progressed in multiple phase II trials in several cancer subtypes and combinations [17]. Based on results from the EMBRACE trial, eribulin got approval as a third-line therapy for patients with MBC who had a prior treatment with an anthracycline and a taxane by the U.S. Food and Drug Administration on 15 November 2010 [15] and by the European Commission on 17 March 2011 [101]. In June 2014, it was approved as a second-line treatment for breast cancer in Europe. It is now approved in 58 countries and as a second-line treatment in 40 countries [102]. However, eribulin was rejected by National Institute of Health and Clinical Excellence in the United Kingdom as it was not considered as a cost-effective use of resources and its failure to demonstrate an extension to life of at least an additional three months in the overall intent to treat population as compared to TPC [103]. However, eribulin is a promising new treatment option in patients with solid tumors and results of other phase I/II/III trials and novel combinations [17] are worth waiting. A list of currently open studies with eribulin is presented in Table 12.

The recommended dose of eribulin mesylate in normal, mild hepatic (Child Pugh A), moderate hepatic (Child Pugh B), and moderate renal impairment (CrCl 30–50 mL/min) is 1.4, 1.1, 0.7 and 1.1 mg/m^2^ respectively, as a 2–5 min i.v. bolus on days 1 and 8 of 21-day cycle [15]. The main treatment-related adverse effects are neutropenia, anemia, fatigue, alopecia, peripheral neuropathy, nausea, and constipation, which are manageable [15]. The most common cause of drug discontinuation is peripheral neuropathy [15].

Liposomal formulation (E7389-LF) [104,105] and novel second-generation analogs which have low P glycoprotein susceptibility [105,106], orally bioavailability, and have blood-brain barrier penetration are currently undergoing development [106]. Different strategies to overcome resistance to eribulin, like multidrug resistance protein 1 inhibitor encapsulation within a nanoparticle delivery system, are being tried in preclinical models [107]. Gene expression profiling to predict eribulin sensitivity is being researched, with epithelial-mesenchymal transition genes showing productivity in breast and endometrial cancer panels [108].

**Table 12:** Currently open studies with eribulin [17].

Study Title	Phase		Sponsors/Collaborators	NCT Number
Study of Eribulin in Children With Cancer to Determine Safety	1		University of Oklahoma	NCT02082626
Phase Ib/II Study of PLX 3397 and Eribulin in Patients With Metastatic Breast Cancer	1/2		Susan G. Komen Breast Cancer Foundation; Plexxikon; University of California, San Francisco	NCT01596751
Pharmacogenomic Study of Neoadjuvant Eribulin for HER2 Non-overexpressing Breast Cancer	2		SOLTI Breast Cancer Research Group; Eisai Inc.	NCT01669252
Neuropharmacokinetics of Eribulin Mesylate in Patients With Brain Metastases From Breast, Bladder, or Non-small Cell Lung Cancer	Not reported		City of Hope Medica Center; National Cancer Institute (NCI); Eisai Inc.	NCT02338037
Eribulin Mesylate and Everolimus in Treating Patients With Triple-Negative Metastatic Breast Cancer	1		City of Hope Medical Center; National Cancer Institute (NCI)	NCT02120469
Eribulin Mesylate in Treating Patients With Locally Advanced or Metastatic Cancer of the Urothelium and Kidney Dysfunction	1/2		National Cancer Institute (NCI)	NCT00365157
An Open-label, Multicenter, Multiple Dose, Phase I Study to Establish the Maximum Tolerated Dose of E7389 Liposomal Formulation in Patients With Solid Tumors	1		Eisai Limited; Eisai Inc	NCT01945710
This is a Phase I Study of Eribulin Mesylate in Pediatric Patients With Recurrent or Refractory Solid Tumors (Excluding CNS), Including Lymphomas.		1	Eisai Inc.	NCT02171260
Combination of Carboplatin, Eribulin Mesylate, and E7449 in BRCA-Related Cancers		1/2	The University of Texas Health Science Center at San Antonio	NCT02396433
Eribulin as 1st Line Treatment in Elderly Patients With Advanced Breast Cancer		2	Swiss Group for Clinical Cancer Research	NCT02404506
Eribulin Mesylate in Treating Patients With Advanced or Recurrent Cervical Cancer		2	University of Southern California; National Cancer Institute (NCI)	NCT01676818
Eribulin Mesylate in Treating Patients With Recurrent or Metastatic Salivary Gland Cancer		2	University of Washington; National Cancer Institute (NCI)	NCT01613768
Eribulin Mesylate in Treating Patients With Previously Treated Metastatic Breast Cancer		2	University of Washington; National Cancer Institute (NCI)	NCT01908101
Eribulin in Combination With Cyclophosphamide in Patients With Solid Tumor Malignancies		1/2	University of California, San Francisco; Eisai Inc.	NCT01554371
Gemcitabine Hydrochloride and Eribulin Mesylate in Treating Patients With Bladder Cancer That is Advanced or Cannot Be Removed by Surgery		2	National Cancer Institute (NCI)	NCT02178241
Eribulin in HER2 Negative Metastatic BrCa		2	Dana-Farber Cancer Institute	NCT01827787
Mifepristone and Eribulin in Patients With Metastatic Triple Negative Breast Cancer or Other Specified Solid Tumors		1	Corcept Therapeutics	NCT02014337
Phase I of Eribulin and Oral Irinotecan for Relapsed or Refractory Solid Tumors		1	University of Kentucky	NCT02318589
Trial of Eribulin Followed by Doxorubicin & Cyclophosphamide for HER2-negative, Locally Advanced Breast Cancer		2	Emory University	NCT01498588
			Eisai Inc.	
Eribulin Plus Gemcitabine (EG) vs Paclitaxel Plus Gemcitabine (PG) in HER2-Negative Metastatic Breast Cancer		2	Asan Medical Center; Eisai Inc.; Dong-A ST Co., Ltd.; Samyang Biopharmaceuticals Corporation	NCT02263495
Safety and Efficacy Study of Eribulin in Combination With Bevacizumab for Second-line Treatment HER2-MBC Patients		2	Consorzio Oncotech	NCT02175446
Selinexor in Combination With Standard Chemotherapy		1	M.D. Anderson Cancer Center; Karyopharm Therapeutics, Inc	NCT02419495
Halaven Post-Marketing Surveillance (PMS)		4	Eisai Korea Inc.; Eisai Inc.	NCT02441764
Phase II Study of Eribulin Mesylate, Trastuzumab, and Pertuzumab in Women With Metastatic, Unresectable Locally Advanced, or Locally Recurrent HER2-Positive Breast Cancer		2	Dana-Farber Cancer Institute; Eisai Inc.; Genentech, Inc.	NCT01912963
A Randomized Phase III Trial of Eribulin Compared to Standard Weekly Paclitaxel as First- or Second-Line Therapy for Locally Recurrent or Metastatic Breast Cancer		3	Academic and Community Cancer Research United	NCT02037529
Neoadjuvant Doxorubicin/Cyclophosphamide Followed by Eribulin Chemotherapy (ACE) in Operable HER2-negative Breast Cancer		2	Sidney Kimmel Comprehensive Cancer Center	NCT02215876
Dose Escalation of POL6326 in Combination With Eribulin in Patients With Metastatic Breast Cancer		1	Polyphor Ltd.	NCT01837095
1303GCC: Trastuzmab & Pertuzumab Alone or in Combination With Hormonal Therapy or Chemotherapy With Eribulin in Women Aged 60 and Over With HER2/Neu Overexpressed Locally Advanced or MBC		2	Genentech, Inc.; University of Maryland	NCT02000596
Retroprospective Real Life Observatory of Eribulin		Not Applicable (Observational)	Institut Cancerologie de l’Ouest	NCT02393287
DETECT IV—A Study in Patients With HER2-negative Metastatic Breast Cancer and Persisting HER2-negative Circulating Tumor Cells (CTCs).		2	University of Ulm	NCT02035813
Safety and Blood Immune Cell Study of Anakinra Plus Physician's Chemotherapy Choice in Metastatic Breast Cancer Patients		1	Baylor Research Institute	NCT01802970
Post-Marketing Surveillance Study of Eribulin on the Status and Factors for the Development of Peripheral Neuropathy in Japan.		4	Eisai Co., Ltd.; Eisai Inc.	NCT02371174
A Study Evaluating Talazoparib (BMN 673), a PARP Inhibitor, in Advanced and/or Metastatic Breast Cancer Patients With BRCA Mutation (EMBRACA Study)		3	BioMarin Pharmaceutical	NCT01945775
			National Breast Cancer Coalition (NBCC)	
			Translational Research in Oncology	
			US Oncology Research	
			Myriad Genetic Laboratories, Inc.	
Assessment of the Efficacy and Safety of Olaparib Monotherapy Versus Physicians Choice Chemotherapy in the Treatment of Metastatic Breast Cancer Patients With Germline BRCA1/2 Mutations. (OlympiAD)		3	AstraZeneca	NCT02000622
			Myriad Genetics—BRAC Analysis test for FDA Premarket Approval (PMA)	
Evaluation of the Efficacy of High Throughput Genome Analysis as a Therapeutic Decision Tool for Patients With Metastatic Breast Cancer (SAFIR02_Breast)		2	UNICANCER	NCT02299999

## References

[1] Uemura D., Takahashi K., Yamamoto T., Katayama C., Tanaka J., Okumura Y., Hirata Y. (1985). Norhalichondrin A: An antitumor polyether macrolide from a marine sponge. J. Am. Chem. Soc..

[2] Hirata Y., Uemura D. (1986). Halichondrins—Antitumor polyether macrolides from a marine sponge. Pure Appl. Chem..

[3] Pettit G.R., Herald C.L., Boyd M.R., Leet J.E., Dufresne C., Doubek D.L., Schmidt J.M., Cerny R.L., Hooper J.N., Rutzler K.C. (1991). Isolation and structure of the cell growth inhibitory constituents from the western pacific marine sponge *Axinella* sp.. J. Med. Chem..

[4] Pettit G.R., Tan R., Gao F., Williams M.D., Doubek D.L., Boyd M.R., Schmidt J.M., Chapuis J.C., Hamel E. (1993). Isolation and structure of Halistatin 1 from the eastern indian ocean marine sponge *Phakellia carteri*. J. Org. Chem..

[5] Gravelos D.G., Lake R., Blunt J.W., Munro M.H.G., Litaudon M.S.P. (1993). Halichondrins: Cytotoxic polyether macrolides. European patent.

[6] Bai R.L., Paull K.D., Herald C.L., Malspeis L., Pettit G.R., Hamel E. (1991). Halichondrin B and Homohalichondrin B, marine natural products binding in the vinca domain of tubulin. Discovery of tubulin-based mechanism of action by analysis of differential cytotoxicity data. J. Biol. Chem..

[7] Towle M.J., Salvato K.A., Budrow J., Wels B.F., Kuznetsov G., Aalfs K.K., Welsh S., Zheng W., Seletsk B.M., Palme M.H. (2001). *In vitro* and *in vivo* anticancer activities of synthetic macrocyclic ketone analogues of Halichondrin B. Cancer Res..

[8] Fodstad O., Breistol K., Pettit G.R., Shoemaker R.H., Boyd M.R. (1996). Comparative antitumor activities of Halichondrins and vinblastine against human tumor xenografts. J. Exp. Ther. Oncol..

[9] Aicher T.D., Buszek K.R., Fang F.G., Forsyth C.J., Jung S.H., Kishi Y., Matelich M.C., Scola P.M., Spero D.M., Yoon S.K. (1992). Total synthesis of Halichondrin B and Norhalichondrin B. J. Am. Chem. Soc..

[10] Yu M.J., Zheng W., Seletsky B.M. (2013). From micrograms to grams: Scale-up synthesis of eribulin mesylate. Nat. Prod. Rep..

[11] Swami U., Chaudhary I., Ghalib M.H., Goel S. (2012). Eribulin—A review of preclinical and clinical studies. Crit. Rev. Oncol. Hematol..

[12] Mani S., Swami U. (2010). Eribulin mesilate, a Halichondrin B analogue, in the treatment of breast cancer. Drugs Today.

[13] Swami U., Shah U., Goel S., Kim S.-K. (2015). Marine Sponge Derived Eribulin in Preclinical and Clinical Studies for Cancer. Handbook of Anticancer Drugs from Marine Origin.

[14] Cortes J., O’Shaughnessy J., Loesch D., Blum J.L., Vahdat L.T., Petrakova K., Chollet P., Manikas A., Dieras V., Delozier T. (2011). Eribulin monotherapy *versus* treatment of physician’s choice in patients with metastatic breast cancer (embrace): A phase III open-label randomised study. Lancet.

[15] U.S. Food and Drug Administration (2010). Highlights of Prescribing Information.

[16] Kaufman P.A., Awada A., Twelves C., Yelle L., Perez E.A., Velikova G., Olivo M.S., He Y., Dutcus C.E., Cortes J. (2015). Phase III open-label randomized study of eribulin mesylate *versus* capecitabine in patients with locally advanced or metastatic breast cancer previously treated with an anthracycline and a taxane. J. Clin. Oncol..

[17] ClinicalTrials.gov. https://clinicaltrials.Gov.

[18] National Cancer Institute, Division of Cancer Treatment and Diagnosis Featured Agents Solicitation for Letters of Intent Clinical Trials Preclinical Experiments. http://dctd.Cancer.Gov/featuredagents/pdfs/e7389solicitationmarch2005.Pdf.

[19] Kimura T., Synold T., Mahaffey C.M., Labauve A.E., Mack P.C., Lenz H.-J., Gandara D.R., Doroshow J.H., Gumerlock P.H. (2003). E7389, a novel antimicrotubule agent with potent p53-independent induction of p27, Bcl2 phosphorylation and cytotoxicity in nonsmall cell lung cancer (NSCLC). Proc. Am. Soc. Clin. Oncol..

[20] Dabydeen D.A., Burnett J.C., Bai R., Verdier-Pinard P., Hickford S.J., Pettit G.R., Blunt J.W., Munro M.H., Gussio R., Hamel E. (2006). Comparison of the activities of the truncated Halichondrin B analog NSC 707389 (E7389) with those of the parent compound and a proposed binding site on tubulin. Mol. Pharmacol..

[21] Alley M.C., Smith A.C., Donohue S.J., Schweikart K.M., Newman D.J., Tomaszewski J.E. Comparison of the Relative Efficacies and Toxicities of Halichondrin B Analogues. Proceedings of the 17th AACR-NCI-EORTC International Conference on Molecular Targets and Cancer Therapeutics.

[22] Budman D.R., Calabro A., Littlefield B.A. Synergestic combinations of E7389 (Halichondrin B analogue) with Conventional Agaents:*In vitro* Median Effect Analysis in Cell Lines with Potential Clinical Implications. Proceedings of the 27th Annual San Antonio Breast Cancer Symposium.

[23] Kurebayashi J., Kanomata N., Yamashita T., Shimo T., Moriya T. (2015). Antitumor and anticancer stem cell activities of eribulin mesylate and antiestrogens in breast cancer cells. Breast Cancer.

[24] Luyimbazi D., Luu T.H., Xing Q., Yan J., Tully D., Han E.S., Yip R., Yim J.H. (2013). Effect of eribulin on cell growth and PI3K pathway activity with and without RAD001 in triple-negative and HER2-expressing breast cancer. J. Clin. Oncol..

[25] Kuznetsov G., Tendyke K., Yu M., Littlefield B.A. Antiproliferative Effects of Halichondrin B Analog Eribulin Mesylate (E7389) Against Paclitaxel-Resistant Human Cancer Cells *in Vitro*. Proceedings of the 2007 AACR-NCI-EORTC International Conference.

[26] Yamaguchi S., Maida Y., Yasukawa M., Kato T., Yoshida M., Masutomi K. (2014). Eribulin mesylate targets human telomerase reverse transcriptase in ovarian cancer cells. PLoS ONE.

[27] LaPointe N.E., Morfini G., Brady S.T., Feinstein S.C., Wilson L., Jordan M.A. (2013). Effects of eribulin, vincristine, paclitaxel and ixabepilone on fast axonal transport and kinesin-1 driven microtubule gliding: Implications for chemotherapy-induced peripheral neuropathy. Neurotoxicology.

[28] Towle M.J., Agoulnik S., Kuznetsov G., TenDyke K., Reardon C., Cheng H., Zheng W., Seletsky B.M., Palme M.H., Kishi Y. (2003). *In vivo* Efficacy of E7389, a Synthetic Analogue of the Marine Sponge Antitubulin Agent Halichondrin B, Against Human Tumor Xenografts Under Monotherapy and Combination Therapy Conditions. Proc. Am. Assoc. Cancer Res..

[29] Towle M.J., Nomoto K., Asano M., Kishi Y., Yu M.J., Littlefield B.A. (2012). Broad spectrum preclinical antitumor activity of eribulin (Halaven^®^): Optimal effectiveness under intermittent dosing conditions. Anticancer Res..

[30] Kolb E.A., Gorlick R., Reynolds C.P., Kang M.H., Carol H., Lock R., Keir S.T., Maris J.M., Billups C.A., Desjardins C. (2013). Initial testing (stage 1) of eribulin, a novel tubulin binding agent, by the pediatric preclinical testing program. Pediatr Blood Cancer.

[31] Wozniak K.M., Nomoto K., Lapidus R.G., Wu Y., Carozzi V., Cavaletti G., Hayakawa K., Hosokawa S., Towle M.J., Littlefield B.A. (2011). Comparison of neuropathy-inducing effects of eribulin mesylate, paclitaxel, and ixabepilone in mice. Cancer Res..

[32] Wozniak K.M., Wu Y., Farah M.H., Littlefield B.A., Nomoto K., Slusher B.S. (2013). Neuropathy-inducing effects of eribulin mesylate *versus* paclitaxel in mice with preexisting neuropathy. Neurotox Res..

[33] Jordan M.A., Kamath K., Manna T., Okouneva T., Miller H.P., Davis C., Littlefield B.A., Wilson L. (2005). The primary antimitotic mechanism of action of the synthetic Halichondrin E7389 is suppression of microtubule growth. Mol. Cancer Ther..

[34] Okouneva T., Azarenko O., Wilson L., Littlefield B.A., Jordan M.A. (2008). Inhibition of centromere dynamics by eribulin (E7389) during mitotic metaphase. Mol. Cancer Ther..

[35] Okouneva T., Wilson L., Littlefield B.A., Jordan M.A. (2004). E7389 and ER-076349, synthetic Halichondrin B analogs, suppress centromere dynamics in concert with mitotic block. Cancer Res..

[36] Kamath K., Okouneva T., Miller H., Davis C., littlefield B.A., Wilson L., Jordan M.A. (2003). E7389, a synthetic analog of Halichondrin B, suppresses microtubule dynamics in living MCF7 cells by a novel mechanism. Proc. Am. Assoc. Cancer. Res..

[37] Smith J.A., Wilson L., Azarenko O., Zhu X., Lewis B.M., Littlefield B.A., Jordan M.A. (2010). Eribulin binds at microtubule ends to a single site on tubulin to suppress dynamic instability. Biochemistry.

[38] Alday P.H., Correia J.J. (2009). Macromolecular interaction of Halichondrin B analogues eribulin (E7389) and ER-076349 with tubulin by analytical ultracentrifugation. Biochemistry.

[39] Kuznetsov G., Towle M.J., Cheng H., Kawamura T., TenDyke K., Liu D., Kishi Y., Yu M.J., Littlefield B.A. (2004). Induction of morphological and biochemical apoptosis following prolonged mitotic blockage by Halichondrin B macrocyclic ketone analog E7389. Cancer Res..

[40] Agoulnik S., Kuznetsov G., Tendyke K., Parent L.A., Marsh J.P., Twine N., Renshaw F.G., Silberman S., Littlefield B.A. Sensitivity to Halichondrin analog E7389 and hemiasterlin analog E7974 correlates with βIII tubulin isotype expression in human breast cancer cell lines. Proceedings of the ASCO Annual Meeting.

[41] Stengel C., Newman S.P., Leese M.P., Potter B.V., Reed M.J., Purohit A. (2010). Class III β-Tubulin expression and *in vitro* resistance to microtubule targeting agents. Br. J. Cancer.

[42] Saussede-Aim J., Matera E.L., Herveau S., Rouault J.P., Ferlini C., Dumontet C. (2009). Vinorelbine induces β3-Tubulin gene expression through an AP-1 site. Anticancer Res..

[43] Seve P., Dumontet C. (2008). Is class III β-tubulin a predictive factor in patients receiving tubulin-binding agents?. Lancet Oncol..

[44] Yoshida T., Ozawa Y., Kimura T., Sato Y., Kuznetsov G., Xu S., Uesugi M., Agoulnik S., Taylor N., Funahashi Y. (2014). Eribulin mesilate suppresses experimental metastasis of breast cancer cells by reversing phenotype from epithelial-mesenchymal transition (EMT) to mesenchymal-epithelial transition (MET) states. Br. J. Cancer.

[45] Suzuki H., Hirata Y., Suzuki N., Ihara S., Sakitani K., Kobayashi Y., Kinoshita H., Hayakawa Y., Yamada A., Watabe H. (2015). Characterization of a new small bowel adenocarcinoma cell line and screening of anti-cancer drug against small bowel adenocarcinoma. Am. J. Pathol..

[46] Agoulnik S.I., Kawano S., Taylor N., Oestreicher J., Matsui J., Chow J., Oda Y., Funahashi Y. (2014). Eribulin mesylate exerts specific gene expression changes in pericytes and shortens pericyte-driven capillary network *in vitro*. Vasc. Cell.

[47] Funahashi Y., Okamoto K., Adachi Y., Semba T., Uesugi M., Ozawa Y., Tohyama O., Uehara T., Kimura T., Watanabe H. (2014). Eribulin mesylate reduces tumor microenvironment abnormality by vascular remodeling in preclinical human breast cancer models. Cancer Sci..

[48] Synold T.W., Morgan R.J., Newman E.M., Lenz H.J., Gandara D.R., Colevas A.D., Lewis M.D., Doroshow J.H. A phase I pharmacokinetic and target validation study of the novel anti-tubulin agent E7389: A california cancer consortium trial. Proceedings of the ASCO Annual Meeting.

[49] Synold T.W., Lawrence J., Xi B., Colevas A.D., Lewis M.D., Doroshow J.H. (2003). Human pharmacokinetics of E7389 (Halichondrin B analog), a novel anti-microtubule agent undergoing phase I investigation in the california cancer consortium (CCC). Proc. Am. Assoc. Cancer Res..

[50] Goel S., Mita A.C., Mita M., Rowinsky E.K., Chu Q.S., Wong N., Desjardins C., Fang F., Jansen M., Shuster D.E. (2009). A phase I study of eribulin mesylate (E7389), a mechanistically novel inhibitor of microtubule dynamics, in patients with advanced solid malignancies. Clin. Cancer Res..

[51] Devriese L.A., Witteveen P.O., Marchetti S., Mergui-Roelvink M., Reyderman L., Wanders J., Jenner A., Edwards G., Beijnen J.H., Voest E.E. (2012). Pharmacokinetics of eribulin mesylate in patients with solid tumors and hepatic impairment. Cancer Chemother. Pharm..

[52] Tan A.R., Sarantopoulos J., Lee L., Reyderman L., He Y., Olivo M.S., Goel S. Pharmacokinetics (PK) of Eribulin Mesylate in Cancer Patients (Pts) with Normal and Impaired Renal Function. Proceedings of the ASCO Annual Meeting.

[53] Devriese L.A., Mergui-Roelvink M., Wanders J., Jenner A., Edwards G., Reyderman L., Copalu W., Peng F., Marchetti S., Beijnen J.H. (2013). Eribulin mesylate pharmacokinetics in patients with solid tumors receiving repeated oral ketoconazole. Invest. New Drugs.

[54] Devriese L.A., Witteveen P.E., Wanders J., Law K., Edwards G., Reyderman L., Copalu W., Peng F., Marchetti S., Beijnen J.H. (2013). Pharmacokinetics of eribulin mesylate in patients with solid tumours receiving repeated oral rifampicin. Br. J. Clin. Pharmacol..

[55] Jansen M., Vernaz-Gris M., DesJardins C., Wong N., Campone M., Cortes J., Wanders J., Shuster D., Fuseau E. Population pharmacokinetics (PPK) of eribulin mesylate in patients with locally advanced or metastatic breast cancer (MBC). Proceedings of the ASCO Annual Meeting.

[56] Dubbelman A.C., Rosing H., Jansen R.S., Mergui-Roelvink M., Huitema A.D., Koetz B., Lymboura M., Reyderman L., Lopez-Anaya A., Schellens J.H. (2012). Mass balance study of [^14^C] eribulin in patients with advanced solid tumors. Drug Metab. Dispos..

[57] Zheng W., Seletsky B.M., Palme M.H. Structure-Activity Relationships of Synthetic Halichondrin B Analog E7389: *In vitro* Susceptibility to PGP-Mediated Drug Efflux. Proceedings of the Annual Meeting Of The American Association For Cancer Research.

[58] Zhang Z.Y., King B.M., Pelletier R.D., Wong Y.N. (2008). Delineation of the interactions between the chemotherapeutic agent eribulin mesylate (E7389) and human Cyp3A4. Cancer Chemother. Pharmacol..

[59] Tan A.R., Rubin E.H., Walton D.C., Shuster D.E., Wong Y.N., Fang F., Ashworth S., Rosen L.S. (2009). Phase I study of eribulin mesylate administered once every 21 days in patients with advanced solid tumors. Clin. Cancer Res..

[60] Mukohara T., Nagai S., Mukai H., Namiki M., Minami H. (2012). Eribulin mesylate in patients with refractory cancers: A phase I study. Invest. New Drugs.

[61] Synold T.W., Tsao-Wei D.D., Quinn D.I., Groshen S.G., Aparicio A., Twardowski P., Stadler W.M., Gandara D.R., Lara P., Newman E.M. (2010). Phase I and Pharmacokinetic (PK) study of eribulin (E7389) in patients (PTS) with renal dysfunction (RD) and advanced urothelial cancer (UC): A california cancer consortium trial. J. Clin. Oncol..

[62] Lesimple T., Edeline J., Carrothers T.J., Cvitkovic F., Darpo B., Delord J.P., Lena H., Penel N., Edwards G.J., Law K. (2013). A phase I, open-label, single-arm study for qt assessment of eribulin mesylate in patients with advanced solid tumors. Invest. New Drugs.

[63] Goel R., Chen E., Welch S., Laurie S., Siu L., Jonker D., Srinivasan R., Wang L., Ivy P., Oza A. (2009). Phase I study of E7389/gemcitabine combination in patients with advanced solid tumors. J. Clin. Oncol..

[64] Koczywas M., Frankel P.H., Synold T.W., Lenz H.J., Mortimer J.E., El-Khoueiry A.B., Gandara D.R., Cristea M.C., Chung V.M., Lim D. (2014). Phase I study of the Halichondrin B analogue eribulin mesylate in combination with cisplatin in advanced solid tumors. Br. J. Cancer.

[65] Mukai H., Saeki T., Shimada K., Naito Y., Matsubara N., Nakanishi T., Obaishi H., Namiki M., Sasaki Y. (2015). Phase I combination study of eribulin mesylate with trastuzumab for advanced or recurrent human epidermal growth factor receptor 2 positive breast cancer. Invest. New Drugs.

[66] Swami U., Petrylak D.P., Raftopoulos H., Shuster D.E., Wang G., Kumar V., Martinez G., Goel S., Aisner J. Phase Ib study of eribulin mesylate in combination with carboplatin in patients with advanced solid tumors. Proceedings of the ASCO Annual Meeting.

[67] Truong T.-G., Pelayo M., Grabowsky J.A., Melisko M.E., Magbanua M.J.M., Moasser M.M., Reinert A., Hwang J., Park J.W., Munster P.N. (2013). Phase Ib study of eribulin (Erb) and cyclophosphamide (Ctx) in metastatic breast cancer (Mbc). J. Clin. Oncol..

[68] Nasim M.Y., Plummer R., Evans T.R.J., Morrison R., Anthoney D.A., Haney S., Madi A., Savulsky C.I., Johnston C., Carter D. (2012). A phase Ib dose-escalation study of eribulin mesylate in combination with capecitabine in patients with advanced/metastatic cancer. J. Clin. Oncol..

[69] Vogelzang N.J., Conkling P., Duran I., Maroto J.P., Modiano M., De Leonardis P., Hodge J.P., Lieberman R. (2012). Phase Ib/II study of eribulin mesylate administered in combination with gemcitabine/cisplatin as first-line therapy for locally advanced or metastatic bladder cancer: Phase Ib results. J. Clin. Oncol..

[70] Sakiyama T., Tsurutani J., Iwasa T., Kawakami H., Nonagase Y., Yoshida T., Tanaka K., Fujisaka Y., Kurata T., Komoike Y. (2015). A phase I dose-escalation study of eribulin and S-1 for metastatic breast cancer. Br. J. Cancer.

[71] Waller C.F., Vynnychenko I., Bondarenko I., Shparyk Y., Hodge J.P., Freeman A., Huber B., Lieberman R., Shelton M.J., Dave H. (2014). An open-label, multicenter, randomized phase Ib/II study of eribulin mesylate administered in combination with pemetrexed *versus* pemetrexed alone as second-line therapy in patients with advanced nonsquamous non-small-cell lung cancer. Clin. Lung Cancer.

[72] Schwartzberg L.S., Tauer K.W., Hermann R.C., Nikolinakos P.G., Houts A.C. (2014). Phase I/II study of neoadjuvant eribulin mesylate, carboplatin, and trastuzumab (ECH) for operable HER2 positive (HER2+) breast cancer. J. Clin. Oncol..

[73] Simon R., Rubinstein L., Arbuck S.G., Christian M.C., Freidlin B., Collins J. (1997). Accelerated titration designs for phase I clinical trials in oncology. J. Natl. Cancer Inst..

[74] Moore M.J., Tang P., Renouf D., Major P., Hedley D., Paterson V., Wang L., Dhesy-Thind B., Southwood B., Doyle L. (2009). A phase II study of Halichondrin B analog eribulin mesylate (E7389) as second-line therapy for patients with advanced pancreatic cancer. J. Clin. Oncol..

[75] Raftopoulos H., Aisner J., Kumar K., Goel S., Dittrich C., Jain M., Gopalakrishna P., Salazar P., Jones B., Petrylak D.P. (2013). Phase Ib extension study of eribulin mesylate in combination with carboplatin in patients with chemotherapy-naive advanced non-small cell lung cancer (NSCLC). J. Clin. Oncol..

[76] Vahdat L.T., Pruitt B., Fabian C.J., Rivera R.R., Smith D.A., Tan-Chiu E., Wright J., Tan A.R., Dacosta N.A., Chuang E. (2009). Phase ii study of eribulin mesylate, a Halichondrin B analog, in patients with metastatic breast cancer previously treated with an anthracycline and a taxane. J. Clin. Oncol..

[77] Cortes J., Vahdat L., Blum J.L., Twelves C., Campone M., Roche H., Bachelot T., Awada A., Paridaens R., Goncalves A. (2010). Phase II study of the Halichondrin B analog eribulin mesylate in patients with locally advanced or metastatic breast cancer previously treated with an anthracycline, a taxane, and capecitabine. J. Clin. Oncol..

[78] Aogi K., Iwata H., Masuda N., Mukai H., Yoshida M., Rai Y., Taguchi K., Sasaki Y., Takashima S. (2012). A phase II study of eribulin in japanese patients with heavily pretreated metastatic breast cancer. Ann. Oncol..

[79] McIntyre K., O’Shaughnessy J., Schwartzberg L., Gluck S., Berrak E., Song J.X., Cox D., Vahdat L.T. (2014). Phase II study of eribulin mesylate as first-line therapy for locally recurrent or metastatic human epidermal growth factor receptor 2-negative breast cancer. Breast Cancer Res. Treat..

[80] Smith J.W., Rege J., Maniar H., Song J., Cox D., O’Shaughnessy J. (2013). Eribulin mesylate (Erib) plus capecitabine (X) for adjuvant treatment in post-menopausal estrogen receptor-positive (ER+) early-stage breast cancer: Phase II, multicenter, single-arm study. J. Clin. Oncol..

[81] Vahdat L.T., Garcia A.A., Vogel C., Pellegrino C., Lindquist D.L., Iannotti N., Gopalakrishna P., Sparano J.A. (2013). Eribulin mesylate *versus* ixabepilone in patients with metastatic breast cancer: A randomized phase II study comparing the incidence of peripheral neuropathy. Breast Cancer Res. Treat..

[82] Yardley D.A., Richards P.D., Reeves J.A., Dees E.C., Osborne C.R.C., Soliman H.H., Paul D., Ademuyiwa F.O., Guthrie T.H., Bromund J.L. (2014). Final results of a phase II study of ramucirumab (RAM) plus eribulin (E) *versus* E in advanced metastatic breast cancer (MBC). J. Clin. Oncol..

[83] Wilks S., Puhalla S., O’Shaughnessy J., Schwartzberg L., Berrak E., Song J., Cox D., Vahdat L. (2014). Phase II, multicenter, single-arm study of eribulin mesylate with trastuzumab as first-line therapy for locally recurrent or metastatic HER2-positive breast cancer. Clin. Breast Cancer.

[84] Abraham J., Robidoux A., Tan A.R., Buyse M.E., Wolmark N., Jacobs S.A. (2014). Phase II randomized clinical trial evaluating neoadjuvant chemotherapy regimens with weekly paclitaxel (WP) or eribulin (E) followed by doxorubicin and cyclophosphamide (AC) in women with locally advanced HER2-negative breast cancer (LABC): Nsabp FB-9. J. Clin. Oncol..

[85] Chalasani P., Robert L.B., Rado T.A., Gadi V.K., Kummet T.D., Specht J.M., Stopeck A., Linden H.M. (2015). Abstract OT2-2-05: Metronomic eribulin in metastatic breast cancer. Cancer Res..

[86] Spira A.I., Iannotti N.O., Savin M.A., Neubauer M., Gabrail N.Y., Yanagihara R.H., Zang E.A., Cole P.E., Shuster D., Das A. (2012). A phase II study of eribulin mesylate (E7389) in patients with advanced, previously treated non-small-cell lung cancer. Clin. Lung Cancer.

[87] Gitlitz B.J., Tsao-Wei D.D., Groshen S., Davies A., Koczywas M., Belani C.P., Argiris A., Ramalingam S., Vokes E.E., Edelman M. (2012). A phase II study of Halichondrin B analog eribulin mesylate (E7389) in patients with advanced non-small cell lung cancer previously treated with a taxane: A california cancer consortium trial. J. Thorac. Oncol..

[88] Mok T.S., Geater S.L., Iannotti N., Thongprasert S., Spira A., Smith D., Lee V., Lim W.T., Reyderman L., Wang B. (2014). Randomized phase II study of two intercalated combinations of eribulin mesylate and erlotinib in patients with previously treated advanced non-small-cell lung cancer. Ann. Oncol..

[89] De Bono J.S., Molife L.R., Sonpavde G., Maroto J.P., Calvo E., Cartwright T.H., Loesch D.M., Feit K., Das A., Zang E.A. (2012). Phase II study of eribulin mesylate (E7389) in patients with metastatic castration-resistant prostate cancer stratified by prior taxane therapy. Ann. Oncol..

[90] Stein M.N., Chen Y., Hudes G.R., Carducci M.A., Tan W., DiPaola R.S. (2010). Ecog 5805: A phase II study of eribulin mesylate (E7389) in patients (Pts) with metastatic castration-resistant prostate cancer (Crpc). J. Clin. Oncol..

[91] Hensley M.L., Kravetz S., Jia X., Iasonos A., Tew W., Pereira L., Sabbatini P., Whalen C., Aghajanian C.A., Zarwan C. (2012). Eribulin mesylate (Halichondrin B analog E7389) in platinum-resistant and platinum-sensitive ovarian cancer: A 2-cohort, phase II study. Cancer.

[92] Ikeda Y., Takano M., Kouta H., Sasaki N., Ikeda S., Kudoh K., Kita T., Kikuchi R., Goto T., Furuya K. (2014). Weekly administration of bevacizumab, eribulin, and oxalilplatin in patients with platinum-resistant and refractory ovarian carcinomas: A phase II study. J. Clin. Oncol..

[93] Schoffski P., Ray-Coquard I.L., Cioffi A., Bui N.B., Bauer S., Hartmann J.T., Krarup-Hansen A., Grunwald V., Sciot R., Dumez H. (2011). Activity of eribulin mesylate in patients with soft-tissue sarcoma: A phase II study in four independent histological subtypes. Lancet Oncol..

[94] Naito Y., Kawai A., Araki N., Ozaki T., Sugiura H., Yazawa Y., Morioka H., Matsumine A., Hiraiwa M., Asami S. (2014). Phase II study of eribulin mesylate in patients (Pts) with advanced soft tissue sarcoma (Sts). J. Clin. Oncol..

[95] Renouf D.J., Tang P.A., Major P., Krzyzanowska M.K., Dhesy-Thind B., Goffin J.R., Hedley D., Wang L., Doyle L., Moore M.J. (2012). A phase II study of the Halichondrin B analog eribulin mesylate in gemcitabine refractory advanced pancreatic cancer. Invest. New Drugs.

[96] Quinn D.I., Aparicio A., Tsao-Wei D.D., Groshen S.G., Dorff T.B., Synold T.W., Stadler W.M., Gandara D.R., Lara P., Newman E.M. (2010). Phase II study of eribulin (E7389) in patients (Pts) with advanced urothelial cancer (UC)—Final report: A california cancer consortium-led Nci/Ctep-sponsored trial. J. Clin. Oncol..

[97] Arnold S.M., Moon J., Williamson S.K., Atkins J.N., Ou S.H., Leblanc M., Urba S.G. (2009). Phase II evaluation of eribulin mesylate (E7389, NSC 707389) in patients with metastatic or recurrent squamous cell carcinoma of the head and neck: Southwest oncology group trial S0618. Invest. New Drugs.

[98] Twelves C., Cortes J., Vahdat L., Olivo M., He Y., Kaufman P.A., Awada A. (2014). Efficacy of eribulin in women with metastatic breast cancer: A pooled analysis of two phase III studies. Breast Cancer Res. Treat..

[99] Muss H., Cortes J., Vahdat L.T., Cardoso F., Twelves C., Wanders J., Dutcus C.E., Yang J., Seegobin S., O’Shaughnessy J. (2014). Eribulin monotherapy in patients aged 70 years and older with metastatic breast cancer. Oncologist.

[100] Spigel D.R., Barlesi F., Felip E., Kim J., Olivo M., Nokihara H., Yang J.C., Satouchi M., Katakami N., Iannotti N. (2014). Efficacy and safety of eribulin compared with treatment of physician’s choice (Tpc) in patients with advanced non-small-cell lung cancer (NSCLC): Results from a phase III study: Locally advanced non-small cell lung cancer. Int. J. Radiat. Oncol. Biol. Phys..

[101] Pean E., Klaar S., Berglund E.G., Salmonson T., Borregaard J., Hofland K.F., Ersboll J., Abadie E., Giuliani R., Pignatti F. (2012). The european medicines agency review of eribulin for the treatment of patients with locally advanced or metastatic breast cancer: Summary of the scientific assessment of the committee for medicinal products for human use. Clin. Cancer Res..

[102] Eisai Inc. Major R&D Pipeline In-House R&D Pipeline List. http://www.Eisai.Com/pdf/eir/erepo/epipeline.pdf.

[103] Greenhalgh J., Bagust A., Boland A., Oyee J., Trevor N., Beale S., Dundar Y., Hockenhull J., Proudlove C., O’Reilly S. (2015). Eribulin for the treatment of advanced or metastatic breast cancer: A nice single technology appraisal. Pharm. Econ..

[104] Yu Y., Desjardins C., Saxton P., Lai G., Schuck E., Wong Y.N. (2013). Characterization of the pharmacokinetics of a liposomal formulation of eribulin mesylate (E7389) in mice. Int. J. Pharm..

[105] Yu M.J., Zheng W., Tendyke K. (2012). Atom-based enumeration: New eribulin analogues with low susceptibility to P-glycoprotein-mediated drug efflux. Bioorg. Med. Chem. Lett..

[106] Narayan S., Carlson E.M., Cheng H., Du H., Hu Y., Jiang Y., Lewis B.M., Seletsky B.M., Tendyke K., Zhang H. (2011). Novel second generation analogs of eribulin. Part I: Compounds containing a lipophilic C32 side chain overcome P-glycoprotein susceptibility. Bioorg. Med. Chem. Lett..

[107] Laughney A.M., Kim E., Sprachman M.M., Miller M.A., Kohler R.H., Yang K.S., Orth J.D., Mitchison T.J., Weissleder R. (2014). Single-cell pharmacokinetic imaging reveals a therapeutic strategy to overcome drug resistance to the microtubule inhibitor eribulin. Sci. Transl. Med..

[108] Dezso Z., Oestreicher J., Weaver A., Santiago S., Agoulnik S., Chow J., Oda Y., Funahashi Y. (2014). Gene expression profiling reveals epithelial mesenchymal transition (Emt) genes can selectively differentiate eribulin sensitive breast cancer cells. PLoS ONE.

